# Primary, secondary and tertiary prevention of long-term benzodiazepine receptor agonists use in Belgium: a policy Delphi

**DOI:** 10.1186/s13690-025-01580-w

**Published:** 2025-06-23

**Authors:** Pauline Van Ngoc, Beatrice Scholtes, Jean-Luc Belche, Melissa Ceuterick

**Affiliations:** 1 Research Unit of Primary Care and Health, Department of General Medicine, Faculty of Medicine, University of Liège, Liège, Belgium; 2 Hedera, Department of Sociology, Faculty of Political and Social Sciences, Ghent University, Ghent, Belgium

**Keywords:** Benzodiazepines, Z drugs, Policy recommendations, Policy Delphi

## Abstract

**Background:**

The long-term use of benzodiazepine receptor agonists (BZRAs) poses a significant public health challenge in Belgium because of the associated risks of physical and psychological dependence. Despite guidelines recommending short-term use, BZRAs are frequently prescribed beyond the recommended duration, leading to chronic use and associated harm. To address this issue, a policy Delphi study was conducted to assess targeted strategies for preventing long-term BZRA use through the lenses of primary, secondary, and tertiary prevention.

**Methods:**

The study involved a panel of experts, including healthcare professionals and patients, who participated in two rounds of questionnaires to evaluate 27 policy recommendations. These recommendations were assessed for feasibility, support, and importance, and participants were also asked whether the necessary conditions were in place to implement each recommendation. This approach aimed to identify areas of consensus and divergence among participants.

**Results:**

Key findings reveal a strong consensus on the need for awareness campaigns aimed at healthcare professionals and the general public to highlight the risks associated with BZRA withdrawal. There was also significant support for implementing training programs to equip healthcare providers with the skills needed to manage BZRA withdrawal effectively. However, some recommendations, such as increasing remuneration for long follow-up consultations and establishing a peer support "benzo-buddy" system, garnered less agreement, suggesting that these proposals require further refinement.

**Conclusion:**

This study highlights the complexity of addressing long-term BZRA use and advocates for a comprehensive, multifaceted approach. This approach should integrate education, awareness, and tailored healthcare practices to increase prevention efforts. The findings emphasise the importance of coordinated interventions across different levels of prevention to effectively mitigate long-term use on BZRAs in Belgium.

By refining and implementing these strategies, the likelihood of achieving meaningful improvements in the management and reduction of chronic BZRA use could be significantly increased, contributing to better public health outcomes.

**Supplementary Information:**

The online version contains supplementary material available at 10.1186/s13690-025-01580-w.

**Table:** 

Text box 1. Contributions to the literature
• Although benzodiazepine receptor agonists (BZRAs) should be used for 2–4 weeks, they are used for longer periods. Long-term use of BZRAs is a major public health challenge. Measures need to be put in place to prevent the long-term use of BZRA.
• This article assesses targeted strategies for preventing long-term BZRA use through the lenses of primary, secondary, and tertiary prevention that could be implemented in Belgium.

## Background

Owing to their adverse effects, the long-term use of benzodiazepine receptor agonists (BZRAs) represents a significant public health concern. These drugs are primarily employed for their anxiolytic and sedative properties. However, their use can result in both short- and long-term adverse effects, including physical and psychological dependence, dizziness, an increased risk of falls, drowsiness, road accidents, and withdrawal difficulties [1–3]. Therefore, guidelines in Belgium recommend that these medications be prescribed for very short periods ranging from 1–4 weeks at the smallest possible dose and as a last resort [4]. Nevertheless, these drugs are widely prescribed and are often used beyond the recommended duration. In fact, a Belgian report showed that the duration of prescriptions was longer than what is recommended in the guidelines, with 67% of participants having been using them for more than 1 year [5].

In this context, the prevention of long-term use of BZRAs becomes crucial and requires a structured, multilevel approach. The concepts of primary, secondary, and tertiary prevention, first articulated by Leavell and Clark [6], provide a foundational framework for addressing health issues at different stages. Primary prevention concentrates on preventing the initial onset of disease by addressing risk factors and promoting healthy behaviours. Secondary prevention aims to identify and treat emerging health problems at an early stage. Tertiary prevention involves action taken after the onset of a disease to minimise complications, prevent further deterioration and improve the quality of life of those affected. When applied to long-term use of BZRAs, primary prevention seeks to avoid unnecessary initial prescriptions, secondary prevention focuses on identifying and reducing long-term use, and tertiary prevention aims to minimise harm in chronic users. These levels of prevention necessitate both specific and coordinated interventions in terms of public health policy and clinical practice.

In Belgium, several policies have been implemented in recent years to address the problem of BZRA use. These policies range from information for patients and the general public, training courses for clinicians and, more recently, a reimbursement programme to help patients taper off the medication. This pilot program launched in February 2023 aimed at reimbursing compounded BZRA preparations for patients undergoing withdrawal [7, 8].

In recent years, Belgium has also undertaken campaigns to prevent the long-term use of BZRAs among both patients and healthcare professionals [9]. A systematic review highlighted that effective public health campaigns need to be well targeted, clearly communicated, and sustained over time to bring about meaningful behavioral change [10]. Some studies have demonstrated that awareness campaigns on medicine use have had limited impacts [10–12], particularly those conducted through social media, which often focus on immediate engagement rather than long-term behavioral change [13]. This underscores the importance of a multilevel approach that incorporates primary, secondary, and tertiary prevention strategies with an evaluation of the impact of these strategies and, furthermore, is adapted to the population it is intended to serve. This article explores various prevention strategies in the context of long-term benzodiazepine receptor agonist use in Belgium, utilising a policy Delphi method to assess and recommend further preventive actions. This approach allows us to explore the opinions of patients, health care professionals and policy-makers familiar with the Belgian context to formulate practical recommendations tailored to Belgium. Our research question is as follows: how can primary, secondary, and tertiary prevention of long-term BZRA use be improved under the current circumstances in Belgium?

## Methods

### Policy Delphi process and recruitment

To answer the research question, a policy Delphi was carried out to establish policy recommendations adapted to current Belgian circumstances. In contrast to the conventional Delphi method, which aims to achieve consensus, the policy Delphi method is intended to uncover the most divergent perspectives and examine a broad spectrum of policy options [14]. This methodology was used to give equal voice to the patients taking part in the study.

The Delphi policy was developed in four phases:

1. **Initial compilations and classifications of recommendations**

Initially, interviews were conducted with a sample of healthcare professionals (*N* = 24) and patients who had either taken or were currently taking BZRA (*N* = 19) (Additional file 1). For the interviews conducted in the initial phase, healthcare professionals and patients were recruited through Belgian mental health and primary care networks, as well as a walloon and Flemish professional medical newspaper. To ensure diversity among healthcare professionals, factors such as geographic location, gender, professional roles, and practice settings were considered, aiming to include at least two participants from each profession (general practitioners, nurses, social workers, psychiatrists, and psychologists) (Additional file 1). For the patient group, long-term BZRA users (≥ 6 months) were targeted, with recruitment through various Belgian mental health networks, primary healthcare channels, social media, and by inviting individuals featured in a French-speaking Belgian television documentary on long-term BZRA use. Eligible patients had prior BZRA experience and had stabilized, reduced, or discontinued usage. A diverse patient sample was ensured by considering variations in experiences, geographic locations, and stages of cessation (Additional file 1).

The interviews with healthcare professionals explored various aspects of managing patients using BZRAs, with questions such as,"What does successful treatment look like to you? "or" In your view, what elements of the Belgian healthcare system (or the regional offer) promote or hinder access to care for typical patients with a substance use disorder related to BZRAs? "The complete interview guide is included in Additional File 2. Similarly, patient interviews focused on their experiences from the initial prescription of BZRAs, their usage trajectory, decisions to stop, stabilize, or reduce use, and the recovery period. At the end of the interview, patients were asked, "What would you do to make this process easier for others, if you could do anything, in an ideal world?" to identify policy recommendations. This interview guide is also included in Additional File 2. All interviews were transcribed and analysed using thematic analysis [15] by the research team (PV, MC, BS) in order to identify policy recommendations. These recommendations underwent thorough discussion and revision within the team to ensure accuracy in their formulation.

Each recommendation was then categorised by the research team (PV, MC, BS) according to prevention tiers via the following definitions [6]:

1. Primary prevention involves strategies aimed at preventing healthy individuals from starting BZRA use by targeting at-risk populations and promoting alternative treatments.
2. Secondary prevention focuses on early detection and intervention to stop the development of long-term BZRA use. Our study included efforts to prevent patients from moving from short-term BZRA prescription to chronic use.
3. Tertiary prevention typically involves rehabilitation; in our study, it involves strategies to reduce and discontinue long-term BZRA use safely while managing any negative effects that may result from prolonged usage.

This structured approach ensured that the policy recommendations were firmly grounded in the experiences and insights of both healthcare professionals and patients.

2. **First round**

For the policy Delphi process, an expert panel consisting of healthcare professionals and patients was assembled via various recruitment strategies. These included a call for participants at a Belgian healthcare conference and the distribution of flyers within the research team's network and through the project’s follow-up committee of stakeholders. Interested individuals were invited to express their initial interest and provide contact information through an online registration form. Participant selection aimed to gather professionals in mental health care, addiction care, primary care, or pharmacy who have a connection to the topic of BZRAs, whether through direct practice or health policy perspectives. For patients, the criterion was having taken or currently taking BZRAs.

A total of 111 participants took part in the first round (see Tables 1 and 2). It took place over two weeks in March 2023 with an online questionnaire via the LimeSurvey platform (Additional file 3). In this first round, the participants completed the online questionnaire. The participants were asked to evaluate (1) the feasibility, and (2) the extent to which they supported each recommendation. Feasibility and support were assessed via a five-point Likert scale ranging from ‘completely disagree’ to ‘completely agree’. The response scales were presented in ascending order to avoid inflated data, acquiescence bias and social desirability bias (the tendency of some respondents to agree with statements or choose positive answers) [16]. The feasibility and support scales were adjusted from Turoff [17], adding a fifth option ‘neither agree nor disagree’. Since these participants were different from those in the initial phase of compiling and identifying policy recommendations, the participants had the opportunity to add additional recommendations in an open text box:"If you have any suggestions for additional recommendations, please indicate them here."

**Table 1:** Sociodemographic data of the patients

	**N**	**%**
**Gender**		
Female	27	84,38
Male	5	15,63
**Age**		
18–40	11	34,38
41–60	14	43,75
> 60	7	21,88
**Regions**		
Brussels	3	9,38
Flanders	25	78,13
Wallonia	4	12,50
**Current occupation among patients**		
Student	2	6,25
Unemployed	3	9,38
Worker	10	31,25
On sick leave/invalid	11	34,38
Retired	4	12,50
Other	2	6,25
**Current BZRA use among patients**		
Using 1 or more BZRA for the long term	6	18,75
In the process of tapering off 1 or more BZRA	5	15,63
Completely tapered off one or more BZRA	16	50
Other	5	15,63
**Round participation**		
Patients who took part in the first round	32	28,83
Patients who took part in the second round	17	27,42

Percentages are calculated based on the total number of patients, except for the"Round Participation"category, where percentages are based on the total number of participants within each round

**Table 2:** Sociodemographic data of the professionals

Responding as	N	%
**Gender**		
Female	50	63,29
Male	29	36,71
**Age**		
18–40	35	44,30
41–60	36	45,57
> 60	8	10,13
**Regions**		
Brussels	20	25,32
Flanders	46	58,23
Wallonia	13	16,46
**Current professions among participants responding as professionals or as a professional who has taken or is taking BZRA**		
General Practitioners	22	27,85
Nurse	1	1,27
Pharmacists	17	21,52
Psychiatrists	8	10,13
Psychologists	11	13,92
Social workers	4	5,06
Other	16	20,25
**Years of experience**		
Between 0–10 years	27	34,18
Between 11–20 years	24	30,38
Between 21- 30 years	16	20,25
> 30 years	12	15,19
**Current BZRA use among professionals who considered themselves as professionals and BZRA users**		
Using 1 or more BZRA for the long term	2	2,53
In the process of tapering off 1 or more BZRA	0	0
Completely tapered off one or more BZRA	4	5,06
Other	0	0
**Round participation**		
Professionals who took part in the first round	79	71,17
Professionals who took part in the second round	45	72,58

Percentages are calculated based on the total number of professionals, except for the"Round Participation"category, where percentages are based on the total number of participants within each round

3. **Analysis of additional recommendations**

The ideas for new policy recommendations put forward by the participants in the first round were reviewed by the team of researchers (BS, MC and PV) to merge the common ideas and remove the elements that were not coherent. This resulted in seven additional policy recommendations, which were revised by the fourth author (JLB) and incorporated into the existing set of 20 recommendations. Seven additional recommendations from the first-round participants were added to the initial 20 derived from interviews with healthcare professionals and patients, resulting in a total of 27 recommendations evaluated in the second round.

4. **Round 2**

The second round took place over two weeks in April 2023 with an online questionnaire via the LimeSurvey platform (Additional file 4). In this second round, 62 participants completed the online questionnaire. During this round, the seven new recommendations that were proposed by participants in the first round were evaluated in terms of (1) feasibility, (2) the extent to which participants supported each recommendation and (3) the importance they assigned to the recommendation. Similarly, in the first round, feasibility and support were assessed via a five-point Likert scale ranging from ‘completely disagree’ to ‘completely agree’. Then, importance was assessed via a five-point Likert Scale from ‘unimportant’ to ‘very important’. A small information box was included to explain the meaning of the importance scale [17]. Additionally, for all 27 recommendations, participants were asked if conditions were met to implement each recommendation with multiple choices: ‘yes’, ‘I don’t know’ and ‘no’. Finally, they were asked to prioritise each recommendation classified per tier of prevention (primary-secondary-tertiary prevention). The participants were asked to select three recommendations among each level of prevention and to rank them in order of importance for implementation given the current circumstances in Belgium.

### Data analysis

The consensus level analysis was conducted on the Policy Delphi based on the technique developed by Meskell et al. [18]. This approach is particularly appropriate as it captures both the extent of consensus and the direction of opinions, offering a more comprehensive understanding of agreement and disagreement. The classification of consensus is based on both agreement scales, providing a structured framework to evaluate consensus levels and the underlying direction of opinions. Responses are categorized into distinct levels of agreement and importance, which are subsequently classified into high, moderate, or low consensus according to predefined thresholds:

### High consensus:

-  ≥ 70% agreement in a single category (completely agree, agree, neither agree nor disagree, disagree, completely disagree OR very important, important, neither important nor unimportant, slightly important, unimportant,).
-  ≥ 80% agreement in grouped categories (completely agree & agree OR disagree & completely disagree OR very important & important OR slightly important & unimportant).

### Moderate consensus:

-  ≥ 60% and < 70% agreement in a single category (completely agree, agree, neither agree nor disagree, disagree, completely disagree OR very important, important, neither important nor unimportant, slightly important, unimportant).
-  ≥ 70% and < 80% agreement in grouped categories (completely agree & agree OR disagree & completely disagree OR very important & important OR slightly important & unimportant).

### Low consensus:

-  > 50% and < 60% agreement in a single category (completely agree, agree, neither agree nor disagree, disagree, completely disagree OR very important, important, neither important nor unimportant, slightly important, unimportant).
-  ≥ 60% and < 70% agreement in grouped categories (completely agree & agree OR disagree & completely disagree OR very important & important OR slightly important & unimportant).

Consensus direction (in favor, against, or neutral) was determined based on the highest level of agreement.

## Results

### Panel characteristics

The policy Delphi panel included 65.8% health professionals, 28.8% patients, and 5.4% considered themselves both. The majority were female (69.4%), aged mainly between 18 and 60 years. Geographically, 21.6% were from Brussels, 53.2% were from Flanders, and 25.2% were from Wallonia. Among professionals, various specialties were represented, with most having 0–10 years of experience. Patients had diverse employment statuses. In terms of their experiences with BZRA, 50% had stopped taking BZRA. Table 1 shows the sociodemographic data of the patients from the expert panel and Table 2 shows the sociodemographic data of the professionals from the expert panel. In the first round, 111 people took part in the online questionnaire. In the second round, 62 participants completed the questionnaire. The response rate was 100% for the first round and 55.85% for the second round.

The study yielded 27 policy recommendations, as shown in Table 3, categorised and aligned with tiers of prevention.

**Table 3:** List of the 27 recommendations

**Primary prevention**		**Initial recommendation (IR) or new recommendation (NR)**
1	Implement an awareness raising campaign among the general public on tapering off BZRA	IR
2	Implement an awareness raising campaign for patients on the challenges of withdrawing BZRA from multiple medications	IR
3	Implement an awareness raising campaign for professionals on the challenges of withdrawing from multiple medications	IR
4	Implement an awareness raising campaign of the risks of BZRA in empathetic and non-stigmatising way	IR
5	Add warnings of the risk of dependence on the BZRA package	IR
6	Undertake further research on the mechanisms surrounding the first prescription of BZRA	NR
**Secondary prevention**		
7	Increase the price per BZRA package	IR
8	Create smaller packages of BZRA	IR
9	Provide information by the prescriber to the patient regarding the risks of dependency of BZRA at first use	IR
10	Provide higher remuneration for prescribers for long follow up consultations dedicated to BZRA	NR
11	Give access to other BZRA prescribers/providers to the part of the medical file related to prescriptions	NR
12	Allow the carer to dispense one or two doses of BZRA at the same time to provide the correct dose	NR
**Tertiary prevention**		
13	Encourage prescribers to add the indication for substance use disorders next to insomnia/anxiety to patient records when use exceeds guidelines	IR
14	Establish an agreement between the prescriber, the pharmacist and the patient to keep the same prescriber and pharmacist throughout treatment	IR
15	Create a shared policy position between different professionals groups in addiction care concerning the management of BZRA	IR
16	Create an inter-professional communication channel at local level, between pharmacists and GPs to discuss common patients	NR
17	Implement a training course on difficult tapering-off processes related to BZRA for professionals	IR
18	Establish and providing a list of healthcare providers specialised in tapering off of BZRA	IR
19	Establish a support and advice line for people who want to taper off of BZRA	IR
20	Develop a ‘Benzo-buddy’ system The'benzo-buddy system'refers to a mentorship and peer support system among individuals with lived experience withdrawing from BZRA (after long-term use)	IR
21	Share patient testimonials about BZRA tapering-off	IR
22	Develop culturally appropriate patient materials	IR
23	Create an ombudsperson for healthcare practitioners to report other practitioners who over-prescribe, prescribe or delivered unsafely BZRA	IR
24	Extend the patient inclusion criteria of the new reimbursement scheme for the compounding of smaller doses of BZRA to residents living in nursing homes	IR
25	Extend the patient inclusion criteria of the new reimbursement scheme for the compounding of smaller doses of BZRA to patients who are taking more than one type of BZRA	IR
26	Offer group therapy to ambulant patients to support the tapering off process	NR
27	Tailor residential addiction care programmes, specifically to BZRA withdrawal	NR

### Level of consensus

Among the recommendations, a level of consensus was established based on the technique developed by Meskell et al. [18] to explore the diversity of agreements and disagreements among the panel of experts in the first and second rounds. To analyse the results, graphs were produced showing the percentage of responses in each response option for each recommendation in terms of its feasibility, the extent to which participants supported each recommendation, the importance they assigned to the recommendation and whether conditions were met to implement each recommendation (Additional file 5). In the first round, percentages were calculated based on the total number of participants (*n* = 111). In the second round, percentages for the seven additional recommendations were calculated based on the number of participants in that round (*n* = 62).

As described in the method, the level of consensus was then calculated and classified using a 4-point scale of high, moderate, low and none (Table 4).

**Table 4:** Level of consensus and direction

Statement number	Feasibility		Support		Importance		Conditions	
	Level of consensus	Direction	Level of consensus	Direction	Level of consensus	Direction	Level of consensus	Direction
Q1	High	+	High	+	High	+	Moderate	+
Q2	High	+	High	+	High	+	Moderate	+
Q3	High	+	High	+	High	+	High	+
Q4	Moderate	+	High	+	High	+	Moderate	+
Q5	High	+	High	+	Moderate	+	Moderate	+
Q6	High	+	High	+	High	+	Low	+
Q7	None		None		Low	-	None	
Q8	Moderate	+	Moderate	+	High	+	Moderate	+
Q9	High	+	High	+	High	+	Moderate	+
Q10	Low	+	None		Low	+	None	
Q11	None		High	+	Moderate	+	Low	=
Q12	Low	+	High	+	Moderate	+	Moderate	+
Q13	Low	+	Low	+	Low	+	None	
Q14	None		Low	+	Low	+	None	
Q15	Moderate	+	High	+	High	+	Low	=
Q16	Moderate	+	High	+	High	+	Low	+
Q17	High	+	High	+	High	+	Moderate	+
Q18	Moderate	+	Moderate	+	Moderate	+	None	
Q19	Low	+	Moderate	+	Low	+	None	
Q20	None		Low	+	None		Low	=
Q21	High	+	High	+	High	+	Low	+
Q22	High	+	High	+	High	+	Low	+
Q23	None		None		None		Low	=
Q24	Low	+	Moderate	+	High	+	None	
Q25	Low	+	Moderate	+	High	+	None	
Q26	Moderate	+	High	+	High	+	None	
Q27	Low	+	Moderate	+	Low	+	None	

### High consensus

Of the 27 recommendations, only one, recommendation 3 (*for the implementation of an awareness-raising campaign for professionals on the challenges of withdrawing from multiple (psychotropic) medications*), was evaluated with a high level of consensus in terms of its feasibility, support, importance and necessary conditions.

Other recommendations have high levels of consensus, with three categories among the categories of ‘feasibility’, ‘support’, ‘importance’ and ‘conditions’, which have a high level of consensus, and only one is considered moderate. These are the following recommendations: number 1 ‘Implement an awareness raising campaign among the general public on tapering off BZRA’; number 2, ‘Implement an awareness raising campaign for patients on the challenges of withdrawing BZRA from multiple medications’; number 9, ‘Provide information by the prescriber to the patient regarding the risks of dependency of BZRA at first use’; and number 17, ‘Implement a training course on difficult tapering-off processes related to BZRA for professionals’.

### Moderate level of consensus

No recommendation was assessed as having a moderate level of consensus for all four categories. Among the recommendations that had moderate consensus in three of the categories, 8 ‘Create smaller packages’ and 18 ‘Establish and provide a list of local healthcare providers trained in tapering off BZRA for healthcare providers and patients’ are recommended. For the recommendations that had 2 categories that were evaluated as a moderate level of consensus, recommendation 4 ‘implement an awareness raising campaign on the risks of BZRA in an empathetic and nonstigmatising way’, recommendation 5 ‘Add warnings of the risks of dependence on packages’ and recommendation 12 ‘Allow the carer to dispense one or two doses of BZRA at a time to provide the correct dose’.

### Low level of consensus

Among the recommendations that were evaluated with a low level of consensus, no recommendation was evaluated with a low consensus for all categories. One recommendation obtained a low level of consensus for 3 categories. This is recommendation 13 ‘encourage prescribers to add the indication for substance use disorders alongside insomnia/anxiety to patient records when use exceeds guidelines’. Among the other recommendations, which have a low level of consensus for two of the four categories, are recommendation 10 ‘Provide higher remuneration for prescribers for long follow-up consultations dedicated to BZRA’, recommendation 14 Establish an agreement between the prescriber, pharmacist, and patient to keep the same prescriber and pharmacist throughout treatment, recommendation 19 ‘Establish a support and advice line for people who want to taper off from BZRA’, recommendation 20 ‘Develop the'benzo-buddy system'and recommendation 27 ‘Tailoring specific residential addiction programs to BZRA’.

### No consensus

None of the recommendations were evaluated with zero consensus in all four categories. Some more restrictive recommendations had three categories with no consensus: recommendation 7 ‘Increase the price per package'and recommendation 23 ‘Create an ombudsperson for healthcare practitioners to report other practitioners who overprescribe or deliver unsafely’. There are also recommendations with 2 categories assessed as having no consensus, but the participants were nonetheless in favour of these recommendations, namely, recommendation 10 ‘Provide higher remuneration for prescribers for long follow-up consultations’, recommendation 14 ‘Establish agreement between prescriber, pharmacists and patients to keep the same prescriber and pharmacists throughout the treatment'and recommendation 20 ‘Develop the benzo-buddy system’.

### Direction of consensus

The direction of the consensus was assessed to indicate whether the consensus was in favour, against or without taking a position (Table 4). There is a direction of consensus only if the level of consensus is high, moderate or low.

With respect to the ‘feasibility’ category, all recommendations with a high, moderate or low level of consensus are included as being in favour of the recommendation, with the exception of recommendation 10, ‘Provide higher remuneration for prescribers for long follow-up consultations’, where the consensus is at the level of the'neither agree nor disagree'response option.

For the ‘support’ category, all the recommendations are considered favourable. For the ‘importance’ category, all are considered favourable, except recommendation 7 ‘Increase the price per BZRA package’. For the ‘conditions’ category, the assessments are favourable to the recommendation, with 4 recommendations where the consensus is in the ‘do not know’ category.

### Disparities in perspectives between patients and healthcare professionals

The level of consensus varied between patients and healthcare professionals for several of the recommendations listed below and in Table 5. There is a jump from one level to another, skipping at least one intermediate stage. For many recommendations, patients did not reach a consensus level, which is noted as ‘none’ whereas for professionals, the level is considered two categories higher (e.g., ‘high’ for professionals and ‘none’ for patients). This is the case for the following items: Feasibility: Q8; Support: Q8, Q14, Q21; Importance: Q1–Q4; Q6; Q8–Q9; Q15–Q18; Q21–Q22; Q24–Q26; Conditions: Q1–Q6; Q8–Q9; Q17; Q22. Additionally, some recommendations stand out. For instance, Recommendation 18 (‘Establish and provide a list of healthcare providers specialized in tapering off BZRA’) shows a low level of consensus among professionals but a high level of consensus among patients regarding feasibility.

**Table 5:** Level of consensus and direction: divergence among professionals and patients

Statement number	Feasibility				Support				Importance				Conditions			
	Professionals		Patients		Professionals		Patients		Professionals		Patients		Professionals		Patients	
	Level of consensus	Direction	Level of consensus	Direction	Level of consensus	Direction	Level of consensus	Direction	Level of consensus	Direction	Level of consensus	Direction	Level of consensus	Direction	Level of consensus	Direction
Q1	High	+	Moderate	+	High	+	High	+	High	+	None		High	+	None	
Q2	High	+	Moderate	+	High	+	High	+	High	+	None		Moderate	+	None	
Q3	High	+	High	+	High	+	High	+	High	+	None		High	+	None	
Q4	High	+	Moderate	+	High	+	High	+	High	+	None		Moderate	+	None	
Q5	High	+	High	+	High	+	High	+	Low	+	None		Moderare	+	None	
Q6	None		None		None		None		High	+	None		Moderate	+	None	
Q7	None		None		None		None		None		None		None		None	
Q8	High	+	None		High	+	None		High	+	None		Moderate	+	None	
Q9	High	+	High	+	High	+	High	+	High	+	None		Moderate	+	None	
Q10	None		None		None		None		None		None		None		None	
Q11	None		None		None		None		Low	+	None		None		None	
Q12	None		None		None		None		Low	+	None		None		None	
Q13	Low	+	None		Low	+	None		None		None		Low	+	None	
Q14	Low	+	None		Moderate	+	None		Low	+	None		None		None	
Q15	Moderate	+	Low	+	High	+	High	+	Moderate	+	None		None		None	
Q16	None		None		Low	+	None		High	+	None		Low	+	None	
Q17	High	+	High	+	High	+	High	+	High	+	None		High	+	None	
Q18	Low	+	High	+	Moderate	+	High	+	Moderate	+	None		None		None	
Q19	Low	+	Moderate	+	Moderate	+	High	+	None		None		None		None	
Q20	None		Low	+	Low	+	Moderate	+	None		None		None		None	
Q21	High	+	Moderate	+	High	+	None	+	Moderate	+	None		Low	+	None	
Q22	High	+	Moderate	+	High	+	High	+	High	+	None		Moderate	+	None	
Q23	None		None	+	None		None		None		None		None		None	
Q24	Low	+	None	+	Moderate	+	Moderate	+	Moderate	+	None		None		None	
Q25	Moderate	+	Low		High	+	Moderate	+	Moderate	+	None		Low	+	None	
Q26	None		None	+	None		None		High	+	None		None		None	
Q27	None		None	+	None		None		Low	+	None		None		None	

A different pattern emerged for Recommendation 20 (‘Develop the'benzo-buddy system’). While patients reached a moderate level of consensus regarding support and a low level of consensus regarding feasibility, healthcare professionals showed low or no consensus for both aspects.

### Prioritisation of recommendations per level of prevention

The participants were invited to select and rank the most important recommendations to implement in the current circumstances. Among the class recommendations at the primary prevention level, recommendation 4, ‘Implementing an awareness raising campaign of the risks of BZRA in an empathetic and nonstigmatising way’, was cited 22 times in the first position. For secondary prevention, recommendation 9, ‘Provide information by the prescriber to the patient regarding the risks of dependency of BZRA at first use’, was cited 22 times. For tertiary prevention, recommendation 17, ‘Implementing a training course on difficult tapering off processes related to BZRA for professionals’, was cited 11 times.

## Discussion

Twenty-seven policy recommendations were evaluated on the basis of their feasibility, support, importance, and conditions required for implementation. Among them, only one recommendation, which focused on raising awareness among professionals about the challenges of withdrawing from multiple psychotropic medications, achieved a high level of consensus across all evaluation categories. Other recommendations, such as those aimed at public awareness, patient education, and professional training, also garnered strong consensus in most categories. However, some showed varying degrees of agreement, with certain recommendations reaching only moderate or low consensus across different areas, indicating, for example, high support but low feasibility due to a lack of necessary conditions present.

The strong support for awareness-raising campaigns suggests that participants view these initiatives as both necessary and currently underutilized in Belgium. Internationally, public health campaigns have been deployed to address benzodiazepine dependence, yet few have been rigorously evaluated for their effectiveness. In Australia, for instance, a multi-strategic approach was implemented to reduce benzodiazepine use, combining media outreach (newspaper articles, radio commercials, posters) with targeted interventions. This has illustrated that sustainable reductions in BZRAs consumption are achievable when awareness initiatives are integrated into broader, evidence-based strategies [19].

Other initiatives, such as the"Choosing Wisely"campaign, have also emerged. Launched in the United States in 2012, the campaign was later adopted in Canada and several European countries [20–23]. Its primary objective was to reduce the use of low-value medical interventions. Within this framework, physicians and patients were encouraged to avoid prescribing benzodiazepines as a first-line treatment for insomnia in older adults. However, no formal evaluation has been conducted on its impact on benzodiazepine prescribing trends, leaving its effectiveness uncertain.

Research shows that well-designed public health initiatives can enhance medication adherence and awareness. However, to be effective across diverse populations, messages must be adapted, clearly formulated, and supported by adequate funding, research, and evaluation [10, 24–28]. In addition, while social media can improve the reach and engagement of health promotion efforts, their impact on long-term behaviour change remains uncertain, highlighting the need for more robust methodologies to measure sustainable outcomes [13].

Given the importance of tailoring awareness campaigns, ensuring that they remain nonstigmatising is equally important, especially when sensitive issues such as BZRA withdrawal are addressed. Stigma, particularly in the context of substance use, can significantly hinder individuals from seeking help and accessing treatment. Research highlights that the language used in public health messaging plays a vital role in either perpetuating or reducing stigma. For instance, terms such as "substance abusers" can evoke punitive attitudes and reinforce negative stereotypes, whereas person-first languages, such as"individuals with a substance use disorder,"help promote more empathetic and supportive public perceptions [29]​. Therefore, any campaign designed to address BZRA withdrawal should prioritise nonstigmatising language, emphasizing support and recovery rather than blame or judgment.

Furthermore, our study demonstrates a high level of consensus on the importance of prescribers providing information to patients about the risks of dependency associated with first use. This aligns with results from another study that highlighted that patients receiving their first prescription for BZRA are often unaware of the potential risks and rarely ask for additional information, relying instead on the trust they place in their physicians [30]. This lack of awareness, coupled with patients'ambiguous feelings about using such medications, underscores the need for prescribers to proactively communicate the risks and benefits from the outset [30]. By setting clear expectations and discussing potential dependency issues early, prescribers can play a crucial role in preventing unintentional long-term use [30]. Additionally, another study underscores the importance of the physician's role in shaping patient attitudes, particularly among patients with little experience with the medication, who are more influenced by their prescriber's perceived norms. This highlights the need for careful consideration of how prescribers'attitudes and advice may impact patients’ medication behaviour and long-term use, ensuring that patients are better equipped to make informed decisions about their treatment [31].

Our results reinforce the need for specialised training programs for professionals on BZRA withdrawal. These proposals were widely endorsed for their feasibility, support and importance, reflecting strong participant approval. In Belgium, several e-learning initiatives on BZRA have already been launched, including the Federal Public Service (SPF) [32], which focuses on patient complaints and nonpharmacological approaches. Additionally, the Belgian Centre for Pharmacotherapeutic Information (CBIP) [33] has introduced a specific training course on benzodiazepine withdrawal for pharmacists and doctors as part of a broader campaign promoting the responsible use of psychotropic drugs. The recommendation for additional training on benzodiazepine withdrawal, despite existing programs in Belgium, may highlight the need for more comprehensive support for healthcare professionals, ensuring that they are well equipped with up-to-date knowledge, practical skills, and tailored guidance to effectively assist patients through the withdrawal process. This finding may also indicate dissatisfaction with the current training courses offered.

On the other hand, certain policy recommendations were characterised by a low level of consensus, such as those aimed at better remunerating prescribers for long follow-up consultations; encouraging prescribers to add the indication for substance use disorders to the record; establishing a tripartite agreement to keep the same prescriber and pharmacist; and developing a ‘benzo-buddy’ system, which is the subject of low consensus in multiple categories, including feasibility, support, importance and conditions. The latter, in particular, received low consensus across multiple criteria, including feasibility, support, importance, and implementation conditions. However, when considering only the perspective of patients—the primary stakeholders of this recommendation—the level of consensus was moderate. This highlights the importance of further exploring such initiatives, as their innovative and original nature may lead them to be overlooked despite their potential impact.

However, insights from studies suggest that adopting a more patient-centered approach, particularly through shared decision-making and the collaborative definition of treatment goals [34, 35], could enhance the effectiveness and acceptance of such strategies. They emphasise the importance of aligning treatment plans with patient expectations and needs, which may address some of the concerns that led to the lower levels of support observed in our study [34, 35].

In addition, other recommendations, such as increasing the price of BZRA packaging, received low consensus, and participants were in disfavour of this recommendation. Creating an ombudsperson for healthcare practitioners to report unsafe practices is a source of debate among the participants. There was no consensus on feasibility, importance or support, and participants did not take a position in favour or in disfavour. Prioritising widely supported recommendations is advisable, as they are more feasible and broadly endorsed. Meanwhile, lower-consensus proposals may need further refinement to address concerns related to implementation. It is also important to be able to bring forward recommendations that may not have had a high level of consensus but that stand out because of their originality, which means that they are less obvious to implement but could be just as effective and inspiring.

### Strengths and limitations

This study has several strengths that contribute to the robustness and relevance of its findings. First, the use of the policy Delphi method allowed for the inclusion of diverse perspectives from both healthcare professionals and patients, which enhanced the comprehensiveness of the policy recommendations. This method also facilitated the identification of areas of consensus and disagreement, providing a nuanced understanding of the complex issue of long-term BZRA use. Additionally, the study's multilevel approach, which focuses on primary, secondary, and tertiary prevention, ensures that the recommendations are well rounded and address the problem from multiple angles.

However, the study also has several limitations. The response rate decreased between the first and second rounds of the Delphi process, which may have impacted the overall consensus and representativeness of the findings. The self-selected sample could also introduce bias, as those with strong opinions or experiences related to BZRA use might be overrepresented. This discrepancy in participant numbers between the two phases represents a limitation of our study. Specifically, the evaluation of the final seven recommendations was conducted by only 62 participants, whereas the initial 20 recommendations were assessed by 111 participants. Although this shows a considerable drop out between round one and two, the minimum required number of participants for this type of multistakeholder Delphi study as set out by Manyara et al. [36] was nonetheless reached [36]. In addition, the classification of the recommendations into primary, secondary, and tertiary prevention levels may present some limitations. Certain recommendations could reasonably be associated with more than one level of prevention. The classification reflects the research team’s interpretation of each recommendation’s predominant focus, while acknowledging that overlaps between prevention tiers are common and may affect how recommendations are understood or prioritized.

## Conclusion

The diversity of recommendations and the levels of consensus underscore the complexity of addressing long-term BZRA use in Belgium. Nevertheless, a comprehensive approach combining education, raising awareness, and healthcare training, seems feasible and well supported. Key strategies include informing patients, training healthcare providers and fostering a supportive environment for withdrawal. Some recommendations received less consensus but stand out for their originality and innovative nature. Notably, like the benzo-buddy system, these recommendations were more strongly supported by patients. Further research could disentangle this hesitancy of health-care providers. Peer support by patients with lived experience is already well established in mental and addiction care, yet still has to be further explored in care for tapering from prescription medication. Integrating multiple strategies is crucial. Applying implementation science framework, such as the behaviour change wheel and the theoretical domain framework, could help structure effective public health interventions and guide future policy development [37].

## Supplementary Information

Additional file 1.

Additional file 2.

Additional file 3.

Additional file 4.

Additional file 5.

## Data Availability

No datasets were generated or analysed during the current study.
