# Supplementary material for: Primary, secondary and tertiary prevention of long-term benzodiazepine receptor agonists use in Belgium: a policy Delphi
Source: Arch Public Health. 2025 Jun 23;83:164. doi: 10.1186/s13690-025-01580-w (PMC12183905; doi:10.1186/s13690-025-01580-w)
Supplement: Supplementary file 1 — Additional file 1. [file 13690_2025_1580_MOESM1_ESM.docx]

**Additional data 1**

**Characteristics of professional participants**

| **Code** | **Interview date** | **Gender** | **Professional background** | **Type of practice** | **Location** | **Region** |
| --- | --- | --- | --- | --- | --- | --- |
| RESP1 | 14-07-21 | Female | GP | Addiction care | Urban | Flanders |
| RESP2 | 20-09-21 | Male | GP | Addiction care | Urban | Flanders |
| RESP3 | 13-10-21 | Male | social worker | Addiction care | Urban | Flanders |
| RESP4 | 20-10-21 | Male | GP | Addiction care | Urban | Flanders |
| RESP5 | 22-10-21 | Male | Psychiatrist | Addiction care | Urban | Flanders |
| RESP6 | 03-11-21 | Female | GP | Addiction care | Urban | Flanders |
| RESP7 | 04-11-21 | Female | GP | Addiction care | Urban | Flanders |
| RESP8 | 17-11-21 | Female | GP | Primary care | Urban | Flanders |
| RESP9 | 30-11-21 | Female | Psychiatrist | Addiction care | Urban | Flanders |
| RESP10 | 09-12-21 | Female | GP | Primary care | Urban/rural | Flanders |
| RESP11 | 29-12-21 | Female | Nurse | Mental health care | Urban | Flanders |
| RESP12 | 07-01-22 | Male | Psychologist | Mental health care | Urban | Flanders |
| RESP13 | 07-01-22 | Male | Psychiatrist | Mental health care | Urban | Flanders |
| RESP14 | 08-07-21 | Female | GP | Primary care | Rural | Wallonia |
| RESP15 | 05-08-21 | Male | GP | Primary care | Urban | Wallonia |
| RESP16 | 23-08-21 | Female | Psychologist | Addiction care | Urban | Brussels |
| RESP17 | 23-08-21 | Female | GP | Addiction care | Urban | Brussels |
| RESP18 | 06-09-21 | Female | Social worker | Addiction care | Rural | Wallonia |
| RESP19 | 09-09-21 | Female | Nurse | Addiction care | Urban | Brussels |
| RESP20 | 10-09-21 | Male | Psychiatrist | Mental health care | Rural | Wallonia |
| RESP21 | 13-09-21 | Male | GP | Primary care | Urban | Brussels |
| RESP22 | 21-09-21 | Male | Psychologist | Addiction care | Rural | Wallonia |
| RESP23 | 22-09-21 | Male | Psychiatrist | Addiction care | Urban | Brussels |
| RESP24 | 19-11-21 | Female | Social worker | Addiction care | Rural | Wallonia |

**Characteristics of patient participants**

| **Code** | **Interview date** | **Gender** | **Year of birth** | **Professional status** | **Reason for first prescription** | **Region** |
| --- | --- | --- | --- | --- | --- | --- |
| RESP1 | 27-04-22 | M | 1980 | Working | Anxiety | Flanders |
| RESP2 | 22-07-22 | M | 1964 | Unemployed | Lexotan for acute psychosis due to drug abuse | Flanders |
| RESP3 | 29-07-22 | F | 1985 | On sick leave | Insomnia | Flanders |
| RESP4 | 31-08-22 | M | 1989 | On sick leave | Anxiety | Flanders |
| RESP5 | 02-09-22 | M | 1969 | Working | Stress | Flanders |
| RESP6 | 15-12-22 | F | 1971 | Unemployed | Insomnia | Flanders |
| RESP7 | 09-05-22 | F | 1948 | Retired | Anxiety | Wallonia |
| RESP8 | 25-05-22 | F | 1948 | Retired | Pain | Wallonia |
| RESP9 | 07-06-22 | F | 1969 | Working | Sleep | Wallonia |
| RESP10 | 23-06-22 | M | 1970 | Unemployed | Anxiety | Wallonia |
| RESP11 | 11-07-22 | F | 1970 | Working | Anxiety | Wallonia |
| RESP12 | 08-09-22 | M | 1949 | Retired | Sleep | Wallonia |
| RESP13 | 09-09-22 | F | 1961 | Retired | Sleep | Wallonia |
| RESP14 | 14-09-22 | F | 1961 | Working | Sleep and anxiety | Wallonia |
| RESP15 | 16-09-22 | F | 1971 | Working | Anxiety | Brussels |
| RESP16 | 21-09-22 | M | 1950 | Retired | Anxiety | Wallonia |
| RESP17 | 30-09-22 | F | 1986 | Working | Anxiety | Brussels |
| RESP18 | 05-10-22 | F | 1973 | Unemployed | Anxiety | Wallonia |
| RESP19 | 10-10-22 | F | 1976 | Working | Anxiety | Wallonia |
