# Supplementary material for: Primary, secondary and tertiary prevention of long-term benzodiazepine receptor agonists use in Belgium: a policy Delphi
Source: Arch Public Health. 2025 Jun 23;83:164. doi: 10.1186/s13690-025-01580-w (PMC12183905; doi:10.1186/s13690-025-01580-w)
Supplement: Supplementary file 2 — Additional file 2. [file 13690_2025_1580_MOESM2_ESM.docx]

**Additional file 2**. **Interview topic guide for healthcare professionals** **and patients**

**Healthcare professionals**

**A. Conceptualization and diagnosis of substance use disorder (SUD) to benzodiazepines and Z-drugs (BZD/Z**)

1. In your general practice how do you diagnose SUD to BZD/Z drugs?

1.1. What criteria to do you use?

1.1.1.Length of time dependent / dosage

1.2. Does this approach differ from how you diagnose dependence to other substances?

1.3. Do you involve other healthcare professionals in the diagnostic process?

1.3.1.If so who?

**B. Types of patients with BZD/Z SUD**

2. What would you say is a typical patient profile? For SUD to BZD?

2.1. What is their typical trajectory - how do typical patients you care for end up with an SUD to BZD?

2.1.1.Do you have many patients that have multiple addictions?

2.1.2. Do you have many patients that also suffer from mental health problems in addition to their SUD?

**C. Treatment of BZD/Z SUD**

3. What is your general approach to treating SUD to BZD?

3.1. How do you approach the issue with your patients?

3.1.1.Goal oriented care, shared-decision making, patient education…?

3.2. Do you struggle to address the issue with patients?

3.2.1.What stops you?

3.2.2.What encourages you?

3.3. What is your usual course of action?

3.3.1.Tapering-off? Substitution with other medication? Supportive therapies/treatments?

3.4. How do you choose between these options? What influences your choice?

3.5. What are the advantages and disadvantages of each?

3.6. How do you manage relapses?

3.7. How do you see the role of the patient in the decision making?

**D. Vision on successful treatment**

4. What does successful treatment look like to you?

4.1. Do you feel there is a tension between you and colleagues on what successful treatment should look like?

4.2. Is there a tension between you and your patients concerning what successful treatment looks like?

4.2.1.Do they want to reach total abstinence?

4.2.2.Do they feel reaching total abstinence is impossible?

4.3. Is there a tension for you between total abstinence and harm reduction?

**E. Vision on prescription of BZD/Z**

5. How do you view prescription of BZD/ Z drugs?

5.1. Is there ever justification for prescribing BZD?

5.1.1.In what circumstances?

5.1.2.How should prescription be managed? Safeguards?

5.2. Does your institution have a policy concerning prescription for BZD/Z drugs?

5.2.1.Do you agree with it?

5.2.2.How do colleagues within the facility view the policy?

5.2.3.What about other colleagues? Other professions/institutions?

**F. Numbers of patients with a BZD/Z SUD (treated), estimate of successful treatment**

6. How successful do feel treatment is? What proportion of your patients go on to successfully complete treatment?

**G. Estimate of possible treatment gap**

7. How many patients do you feel need treatment but are not getting it?

7.1. Out of 10 patients who need treatment how many do you think are receiving treatment?

**H. Perception of barriers and facilitators / protective factors**

8. In your view what facilitates the access to care for patients in Belgium - protective factors? (Health beliefs, social

8.1. What hinders it? (Financial means of the patient, geography etc.)

8.2. In your view what facilitates the role of the provider in the providing treatment to patients with SUD to BZD?

8.3. What barriers may the provider encounter to providing treatment?

8.4. In your view what elements of the Belgian health care system (or the regional offer) promote access to care among typical patients with a SUD to BZD?

8.4.1.What elements of the system hinder access to care?

**I. Some statistics about your practice**

10. How many patients are you responsible for?

11. What type of practice are you working in?

12. What would you say is the socio-economic situation of patients in your practice? Living in poverty, poor,

comfortable

**J. Final reflective question: on impact of recent reforms in the mental health/ addiction care sector on prescribing/ deprescribing of BZD/Z**

13. How do feel the recent reforms in mental healthcare have impacted the addiction care sector?

13.1. How has this impacted prescribing BZD/Z?

13.2. How has this impacted deprescribing of BZD/Z?

**Patients**

**Topic guide**

**Where are they in the cycle of Prochaska and Diclemente**1**?**

- Are you currently (tick all that apply): completely stopped, actively tapering-off/reducing doses, at a stable dose (no plans to reduce)
- Choose an answer that corresponds to your situation:
- I stopped taking sedatives and/or tranquilizers.
- I started to reduce the number of sedatives and/or tranquilizers.
- I did not plan to reduce the number of sedatives and/or tranquilizers.

**Type of medication**

- What kind(s) of sedatives and/or tranquilizers have you taken regularly?
- What kind(s) of sedatives and/or tranquilizers do you take regularly?

**INTRODUCTORY QUESTION**

Tell me about your experiences **recovering from** using sedatives and/or tranquilizers?

Aim: get basic background history

Pertinent additional questions can be asked, for example to explore overarching concepts and definitions.

**CALENDAR METHOD**

For this question we will employ an adjusted life history calendar method (Nelson, 2010) 2 centered on the participant’s medication and cessation history. The participant will be invited to draw a timeline of his/her medication and cessation history. We will allow the participant to fill this in freely. We will ask them to indicate the following major event points (in line with the different phases outlined by DiClemente and Prochaska, 1998) 1:

- ONSET: starting point of medication (=precontemplation phase)
- USE TRAJECTORY: evolution of medication use (=precontemplation phase)
- DECISION TO STOP/STABILISE OR REDUCE: turning point (contemplation + preparation phase)
- RECOVERY : cessation, stabilisation (action + possible relapse(s) + maintenance phases)

**ONSET**

- When did you start taking the medication?
- What was or were the reason(s) for the first medication? (sleep problems, anxiety, chronic pain,…)
- Who prescribed it? / How do you access your sedatives and/or tranquilizers?
- What were the circumstances in your personal life related to the need to start medication?
  - - Personal life (Children? How old at that time? Marriages ? Deaths ?
    - Professional life? (work?)
    - Stressful life events?

**USE TRAJECTORY**

- How did your use of this medication evolve over time?
- In what dosage did you use it?
- How did you obtain it?
- How did you manage to get more?
- Did you combine this with other substances (e.g. alcohol, medication, drugs,…)? If so, which ones?
- What were the benefits of using this medication for you? What were the positive impacts?
- What were the disadvantages of using of this medication for you?
- What were the interaction with professionals? On your own?

**DECISION TO STOP/STABILISE/REDUCE**

- At what point did was your use was becoming problematic to you? What was the turning point for you?
- At what point did you decide to stop?
- What were the context and reasons behind your decision?
- Who played a role in that (health care professionals)?
- What were your objectives?
- What do you consider to be recovered? What do you consider a satisfactory situation?
- Prompt into how they describe the experience, and take the liberty to go a bit deeper into what they say… How was it to X or Y… Can you elaborate a bit on X or Y.. Give an example of X or Y…

**RECOVERY (stabilization, stop, reduce, satisfactory situation)**

- What was the first step? (Did the decision come from yourself or did someone else suggest it (e.g. Health Care Professional )? Probes (based on BENZONET study3):
  - - What/who helped to take that first step ?
      - alternatives for the underlying anxiety/sleeping problems (sleep therapy in a sleep clinic or through a physiotherapist, psychotherapy, meditation (self-taught through online videos), sports, natural remedies (herbs, melatonin)
      - peer support
      - support of family and informal network
      - change in context and daily schedule (for example due to retirement, change of career,…)
      - moral support of the attending physician
      - recognition of the patient’s experiences during withdrawal are (validation)
      - accessibility to (small) correct taper dosages (taper strips, pharmaceutical preparations)
      - tapering at own pace/rhythm (mutual agreement on treatment)
    - What/who hindered?
      - stigma/shame to seek help
      - no feasible alternatives for the underlying anxiety/sleeping problems
      - unsupportive family and informal network
      - invalidation of withdrawal effects by treating health care professional
      - no accessibility to (small) correct taper dosages (taper strips, pharmaceutical preparations)
      - limited knowledge on support for cessation options
      - when under medical supervision: unrealistic tapering schedule
- At that point, when you decided to seek help, did you experience problems to access the care you felt you needed?
- What were the circumstances in your personal or professional life? (Refer to previous responses)
- Who was supporting you? What exactly was supportive about their help? (Here we will show a list of possible sources of help, accompanied by an icon)
- Pharmacist
- GP
- Psychologist (where did you consult with them)
- Psychiatrist
- Nurse
- Peer support (off-or online), coach
- Informal network: partner, family member, friend…
- Physiotherapist
- Alternative therapist
- Optional question: are you a believer?
- Whose help were you missing?
- How did your recovery evolve? Can you describe the process?
- How did you experience that part of the treatment?
- Physical effects?
- Psychological effects?
- …. ?
- How was your interaction with the health care system during your recovery?
- Can you describe the role of each professional?
- What helped?
- What hindered?
- At what point did you consider yourself to be recovered? And how do you describe recovery?
- what are the benefits and disadvantages of stopping/ to be stabilised?
- Are you currently still doing things to maintain this balance?

**CLOSING QUESTIONS**

- What would you do to make this process easier for others, if you could do anything, in an ideal world?
- Is there anything else you want to mention?

**References**

DiClemente CC and Prochaska JO (1998) Toward a comprehensive, transtheoretical model of change: Stages of change and addictive behaviors. In Miller WR and Heather N (Eds.) Applied clinical psychology. Treating addictive behaviors: 3–24. Plenum Press. https://doi.org/10.1007/978-1-4899-1934-2_1

2 Nelson, I. A. (2010). From quantitative to qualitative: Adapting the life history calendar method. Field methods, 22(4), 413-428.

3 Ceuterick M, Christiaens T, Creupelandt H, Bracke P. Perception, habitual use and cessation of BENZOdiazepines: a multi-method NETnography. Final Report. Brussels : Belgian Science Policy Office 2021 – 104 p. (Federal Research Programme on Drugs
