# Supplementary material for: Primary, secondary and tertiary prevention of long-term benzodiazepine receptor agonists use in Belgium: a policy Delphi
Source: Arch Public Health. 2025 Jun 23;83:164. doi: 10.1186/s13690-025-01580-w (PMC12183905; doi:10.1186/s13690-025-01580-w)
Supplement: Supplementary file 3 — Additional file 3. [file 13690_2025_1580_MOESM3_ESM.html]

LimeSurvey v3.15 - BENZOCARE policy recommendations


# BENZOCARE policy recommendations

Dear Sir/Madam,

The purpose of this survey is to **establish policy recommendations on the managemement of sleeping pills and sedatives (benzodiazepines and Z-drugs)** based on **your experience as a professional** in mental health care, addiction care, primary health care, pharmaceuticals **or as a patient** who has taken or is taking sleeping pills and sedatives. 

Based on interviews with professionals and patients, twenty recommendations emerged. In this questionnaire, we would like you to assess the feasibility and the extent to which you support these proposed recommendations. 

The order of recommendations in the survey follows the trajectory from primary prevention (when a person is not yet using) to tertiary prevention when a person is already using heavily.

Completing the questionnaire will take you approximately 15 minutes.

You will be contacted again to complete **a second questionnaire at the end of April** to further analyse these recommendations. You will be asked what conditions are necessary to make each recommendation feasible and how do you prioritise them. 

Thank you for your collaboration and participation.

There are 62 questions in this survey.

## 

### My participation is voluntary. I have the right to withdraw my consent at any time without giving a reason. \*

Please choose **only one** of the following:

- Yes

### In order to meet the needs of this study, I consent to the collection and use of my data; \*

Please choose **only one** of the following:

- Yes

### I authorise the consultation of my data to the persons collaborating in this research. \*

Please choose **only one** of the following:

- Yes

### I give permission to reuse my data for further scientific research outside the scope of the current study. \*

Please choose **only one** of the following:

- Yes
- No

### After this study, I agree to be approached for further research. \*

Please choose **only one** of the following:

- Yes
- No

### I wish to participate in this survey. \*

Please choose **only one** of the following:

- Yes

## 

### Your name \*

Please write your answer here:

Your details are being collected in order to facilitate the second questionnaire which will be sent to you personally at the end of April. This information will then be coded and pseudonomysed.

### Your firstname \*

Please write your answer here:

Your details are being collected in order to facilitate the second questionnaire which will be sent to you personally at the end of April. This information will then be coded and pseudonomysed.

### Your email address \*

Please write your answer here:

Your email address will only be used for the purposes of this study. Your details are being collected in order to facilitate the second questionnaire which will be sent to you personally at the end of April. This information will then be coded and pseudonomysed.

### What country do you live in?  \*

Please choose **only one** of the following:

- Belgium
- Luxembourg
- Netherlands
- France

### In this study, are you responding as : \*

Please choose **only one** of the following:

- A patient who has taken or is taking sleeping pills or sedatives
- A professional in mental health care, addiction care, primary care or pharmaceutical or a healthcare professional
- Both

### What is your gender ? \*

Please choose **only one** of the following:

- Female
- Male
- Other

### What is year of birth ? \*

Please enter a date:

### What is your current profession ?  \*

Please choose **only one** of the following:

- Psychiatrist
- General practionner
- Psychologist
- Pharmacist
- Nurse
- Social worker
- Health care professional
- Other

### What is the postal code of your principal activity? \*

Please write your answer here:

### How many years of experience do you have in your profession (for physicians: including years as physician assistant)?  \*

Please write your answer here:

in years.

### What is your postcode ?  \*

Please write your answer here:

### What is your occupation? \*

Please choose **only one** of the following:

- Student
- Worker
- On sick leave
- Unemployed
- Retired
- Other

### Have you ever used sleeping sleeping pills or sedatives (benzodiazepines and Z-drugs) longer than 2-4 weeks?  \*

Please choose **only one** of the following:

- Yes
- No

### Are you currently:  \*

Please choose **only one** of the following:

- In the process of tapering-off one or more benzodiazepines and Z-drugs
- Completely tapered-off one or more benzodiazepines and Z-drugs
- Using one or more benzodiazepines and Z-drugs in the long term
- Other

### Are you helping other patients to taper off benzodiazepines and Z-drugs?  \*

Please choose **only one** of the following:

- Yes
- No

## 

### How strongly do you agree or disagree with the following statements? \*

Please choose the appropriate response for each item:

|  | Completely disagree | Disagree | Neither agree nor disagree | Agree | Completely agree |
| --- | --- | --- | --- | --- | --- |
| In the current circumstances, it is **feasible** to implement an awareness raising campaign among the general public on tapering off benzodiazepines and Z-drugs. |  |  |  |  |  |
| In the current circumstances, I would **support** the implementation of an awareness raising campaign among the general publicon tapering off benzodiazepines and Z-drugs. |  |  |  |  |  |

### If you would like to elaborate on your responses, please do so here (optional):

Please write your answer here:

## 

### How strongly do you agree or disagree with the following statements? \*

Please choose the appropriate response for each item:

|  | Completely disagree | Disagree | Neither agree nor disagree | Agree | Completely agree |
| --- | --- | --- | --- | --- | --- |
| I the current circumstances, it is **feasible** to implement an awareness raising campaign for patients on the challenges of withdrawing benzodiazepines and Z-drugs from multiple medications. |  |  |  |  |  |
| I the current circumstances, I would **support** the implementation of an awareness raising campaign for patients on the challenges of withdrawing benzodiazepines and Z-drugs from multiple medications. |  |  |  |  |  |

### If you would like to elaborate on your responses, please do so here (optional):

Please write your answer here:

## 

### How strongly do you agree or disagree with the following statements? \*

Please choose the appropriate response for each item:

|  | Completely disagree (1) | Disagree (2) | Neither agree nor disagree (3) | Agree (4) | Completely agree (5) |
| --- | --- | --- | --- | --- | --- |
| In the current circumstances, it is **feasible** to implement an awareness raising campaign for professionals on the challenges of withdrawing from multiple medications. |  |  |  |  |  |
| In the current circumstances, I would **support** the implementation of an awareness raising campaign for professionalson the challenges of withdrawing from multiple medications. |  |  |  |  |  |

### If you would like to elaborate on your responses, please do so here (optional):

Please write your answer here:

## 

### How strongly do you agree or disagree with the following statements? \*

Please choose the appropriate response for each item:

|  | Completely disagree (1) | Disagree (2) | Neither agree nor disagree (3) | Agree (4) | Completely agree (5) |
| --- | --- | --- | --- | --- | --- |
| In the current circumstances, it is **feasible** to implement an awareness raising campaign of the risks of benzodiazepines and Z-drugs in empathetic and non-stigmatising way. |  |  |  |  |  |
| In the current circumstances, I would **support**an awareness raising campaign of the risks of benzodiazepines and Z-drugs in empathetic and non-stigmatising way. |  |  |  |  |  |

### If you would like to elaborate on your responses, please do so here (optional):

Please write your answer here:

## 

### How strongly do you agree or disagree with the following statements? \*

Please choose the appropriate response for each item:

|  | Completely disagree | Disagree | Neither agree nor disagree | Agree | Completely agree |
| --- | --- | --- | --- | --- | --- |
| In the current circumstances, it is **feasible** to add warnings of the risk of dependance on the benzodiazepines and Z-drugspackage. |  |  |  |  |  |
| In the current circumstances, I would **support** the addition of warnings of the risk of dependance on the benzodiazepines and Z-drugs package. |  |  |  |  |  |

### If you would like to elaborate on your responses, please do so here (optional):

Please write your answer here:

## 

### How strongly do you agree or disagree with the following statements? \*

Please choose the appropriate response for each item:

|  | Completely disagree | Disagree | Neither agree nor disagree | Agree | Completely agree |
| --- | --- | --- | --- | --- | --- |
| In the current circumstances, it is **feasible** to increase the price per benzodiazepines and Z-drugs package. |  |  |  |  |  |
| In the current circumstances, I would **support** increasing the price per benzodiazepines and Z-drugs package. |  |  |  |  |  |

### If you would like to elaborate on your responses, please do so here (optional):

Please write your answer here:

## 

### How strongly do you agree or disagree with the following statements? \*

Please choose the appropriate response for each item:

|  | Completely disagree | Disagree | Neither agree nor disagree | Agree | Completely agree |
| --- | --- | --- | --- | --- | --- |
| In the current circumstances, it is **feasible** to create smaller packages of benzodiazepines and Z-drugs is feasible. |  |  |  |  |  |
| In the current circumstances, I would **support** the creation of smaller packages of benzodiazepines and Z-drugs. |  |  |  |  |  |

### If you would like to elaborate on your responses, please do so here (optional):

Please write your answer here:

## 

### How strongly do you agree or disagree with the following statements?  \*

Please choose the appropriate response for each item:

|  | Completely disagree | Disagree | Neither agree nor disagree | Agree | Completely agree |
| --- | --- | --- | --- | --- | --- |
| In the current circumstances, it is **feasible** to provide information by the prescriber to the patient regarding the risksof dependency of benzodiazepines and Z-drugs at first use. |  |  |  |  |  |
| In the current circumstances, I would **support** the provision of information by the prescriber to the patient regarding the risks of dependency of benzodiazepines and Z-drugs at first use. |  |  |  |  |  |

### If you would like to elaborate on your responses, please do so here (optional) :

Please write your answer here:

## 

### How strongly do you agree or disagree with the following statements? \*

Please choose the appropriate response for each item:

|  | Completely disagree | Disagree | Neither agree nor disagree | Agree | Completely agree |
| --- | --- | --- | --- | --- | --- |
| In the current circumstances, it is **feasible** to encourage prescribers to add the indication for substance use disorders next to insomnia/anxiety to patient records when use exceeds guidelines. |  |  |  |  |  |
| In the current circumstances, I would **support** the encouragement of prescribers to add the indication for substance use disorders next to insomnia/anxiety to patient records when use exceeds guidelines. |  |  |  |  |  |

### If you would like to elaborate on your responses, please do so here (optional):

Please write your answer here:

## 

### How strongly do you agree or disagree with the following statements? \*

Please choose the appropriate response for each item:

|  | Completely disagree | Disagree | Neither agree nor disagree | Agree | Completely agree |
| --- | --- | --- | --- | --- | --- |
| In the current circumstances, it is **feasible** to establish an agreement between the prescriber, the pharmacist and the patient to keep the same prescriber and pharmacist throughout treatment. |  |  |  |  |  |
| In the current circumstances, I would **support** the establishment of an agreement between the prescriber, the pharmacist and the patient to keep the same prescriber and pharmacist throughout treatment. |  |  |  |  |  |

### If you would like to elaborate on your responses, please do so here (optional):

Please write your answer here:

## 

### How strongly do you agree or disagree with the following statements?  \*

Please choose the appropriate response for each item:

|  | Completely disagree | Disagree | Neither agree nor disagree | Agree | Completely agree |
| --- | --- | --- | --- | --- | --- |
| In the current circumstances, it is **feasible** to create a shared policy position between professional groups in addiction care concerning the management of benzodiazepines and Z-drugs. |  |  |  |  |  |
| In the current circumstances, I would support the creation of a shared policy position between professional groups in addiction care concerning the management of benzodiazepines and Z-drugs. |  |  |  |  |  |

### If you would like to elaborate on your responses, please do so here (optional):

Please write your answer here:

## 

### How strongly do you agree or disagree with the following statements? \*

Please choose the appropriate response for each item:

|  | Completely disagree | Disagree | Neither agree nor disagree | Agree | Completely agree |
| --- | --- | --- | --- | --- | --- |
| In the current circumstances, it is **feasible** to implement a training course on difficult tapering-off processes related to benzodiazepines and Z-drugs for professionals. |  |  |  |  |  |
| In the current circumstances, I would **support** the implementation of a training course on difficult tapering-off processes related to benzodiazepines and Z-drugs for professionals. |  |  |  |  |  |

### If you would like to elaborate on your responses, please do so here (optional):

Please write your answer here:

## 

### How strongly do you agree or disagree with the following statements? \*

Please choose the appropriate response for each item:

|  | Completely disagree | Disagree | Neither agree nor disagree | Agree | Completely agree |
| --- | --- | --- | --- | --- | --- |
| In the current circumstances, it is **feasible** to establish and providing a list of local healthcare providers trained in tapering off of benzodiazepines and Z-drugs for healthcare providers and patients. |  |  |  |  |  |
| In the current circumstances, I would **support** the establishment and provision of a list of local healthcare providers trained in tapering off of benzodiazepines and Z-drugs for healthcare providers and patients. |  |  |  |  |  |

### If you would like to elaborate on your responses, please do so here (optional):

Please write your answer here:

## 

### How strongly do you agree or disagree with the following statements? \*

Please choose the appropriate response for each item:

|  | Completely disagree | Disagree | Neither agree nor disagree | Agree | Completely agree |
| --- | --- | --- | --- | --- | --- |
| In the current circumstances, it is **feasible** to establish a support and advice line for people who want to taper off of benzodiazepines and Z-drugs. |  |  |  |  |  |
| In the current circumstances, I would **support** the establishment of a support and advice line for people who want to taper off of benzodiazepines and Z-drugs. |  |  |  |  |  |

### If you would like to elaborate on your responses, please do so here (optional):

Please write your answer here:

## 

### How strongly do you agree or disagree with the following statements? \*

Please choose the appropriate response for each item:

|  | Completely disagree | Disagree | Neither agree nor disagree | Agree | Completely agree |
| --- | --- | --- | --- | --- | --- |
| In the current circumstances, it is **feasible** to develop a ‘Benzo-buddy’ system. |  |  |  |  |  |
| In the current circumstances, I would **support** the development of a 'Benzo buddy' system. |  |  |  |  |  |

### If you would like to elaborate on your responses, please do so here (optional):

Please write your answer here:

## 

### How strongly do you agree or disagree with the following statements? \*

Please choose the appropriate response for each item:

|  | Completely disagree | Disagree | Neither agree nor disagree | Agree | Completely agree |
| --- | --- | --- | --- | --- | --- |
| In the current circumstances, it is **feasible** to share patient testimonials about benzodiazepines and Z-drugs tapering-off. |  |  |  |  |  |
| In the current circumstances, I would **support** the share of patient testimonials about benzodiazepines and Z-drugs tapering-off. |  |  |  |  |  |

### If you would like to elaborate on your responses, please do so here (optional):

Please write your answer here:

## 

### How strongly do you agree or disagree with the following statements? \*

Please choose the appropriate response for each item:

|  | Completely disagree | Disagree | Neither agree nor disagree | Agree | Completely agree |
| --- | --- | --- | --- | --- | --- |
| In the current circumstances, it is **feasible** to develop culturally appropriate patient materials. |  |  |  |  |  |
| In the current circumstances, I would **support** the development of culturally appropriate patient materials. |  |  |  |  |  |

### If you would like to elaborate on your responses, please do so here (optional):

Please write your answer here:

## 

### How strongly do you agree or disagree with the following statements? \*

Please choose the appropriate response for each item:

|  | Completely disagree | Disagree | Neither agree nor disagree | Agree | Completely agree |
| --- | --- | --- | --- | --- | --- |
| In the current circumstances, it is **feasible** to create an ombudsperson for healthcare practitioners to report other practitioners who over-prescribe, prescribe or delivered unsafely. |  |  |  |  |  |
| In the current circumstances, I would **support** the creation of an ombudsperson for healthcare practitioners to report other practitioners who over-prescribe, prescribe or delivered unsafely. |  |  |  |  |  |

### If you would like to elaborate on your responses, please do so here (optional):

Please write your answer here:

## 

### How strongly do you agree or disagree with the following statements? \*

Please choose the appropriate response for each item:

|  | Completely disagree | Disagree | Neither agree nor disagree | Agree | Completely agree |
| --- | --- | --- | --- | --- | --- |
| In the current circumstances, it is **feasible** to extend the patient inclusion criteria of the new reimbursement scheme for the compounding of smaller doses of benzodiazepines and Z-drugs to residents living in nursing homes is feasible. |  |  |  |  |  |
| In the current circumstances, I would **support** the extension of the patient inclusion criteria of the new reimbursement scheme for the compounding of smaller doses of benzodiazepines and Z-drugs to residents living in nursing homes. |  |  |  |  |  |

### If you would like to elaborate on your responses, please do so here (optional):

Please write your answer here:

## 

### How strongly do you agree or disagree with the following statements? \*

Please choose the appropriate response for each item:

|  | Completely disagree | Disagree | Neither agree nor disagree | Agree | Completely agree |
| --- | --- | --- | --- | --- | --- |
| In the current circumstances, it is **feasible** to extend the patient inclusion criteria of the new reimbursement scheme for the compounding of smaller doses of benzodiazepines and Z-drugs to patients who are taking more than one type of benzodiazepines or Z-drugs. |  |  |  |  |  |
| In the current circumstances, I would **support** the extension of patient inclusion criteria of the new reimbursement scheme for the compounding of smaller doses of benzodiazepines and Z-drugs to patients who are taking more than one type of benzodiazepines or Z-drugs. |  |  |  |  |  |

### If you would like to elaborate on your responses, please do so here (optional):

Please write your answer here:

## 

### If you have any suggestions for additional recommendations, please indicate them here:

Please write your answer here:

Thank you for your participation to this study!

You will be contacted again to complete **a second questionnaire at the end of April** to further analyse these recommendations. You will be asked what conditions are necessary to make each recommendation feasible and how do you prioritise them. 

If you have any questions, feel free to email us at dumg@uliege.be 

  
24.03.2023 – 11:17  
  
Submit your survey.  
Thank you for completing this survey.
