# Supplementary material for: Primary, secondary and tertiary prevention of long-term benzodiazepine receptor agonists use in Belgium: a policy Delphi
Source: Arch Public Health. 2025 Jun 23;83:164. doi: 10.1186/s13690-025-01580-w (PMC12183905; doi:10.1186/s13690-025-01580-w)
Supplement: Supplementary file 4 — Additional file 4. [file 13690_2025_1580_MOESM4_ESM.html]

LimeSurvey v3.15 - BENZOCARE policy recommendations - second round


# BENZOCARE policy recommendations - second round

Dear Sir/Madam,

We would like to thank you for completing the first survey for the BENZOCARE study. We received a lot of valuable feedback on the different recommendations with regard to benzodiazepines and Z-drugs, as well as suggestions for new additional recommendations.

We now kindly invite you to participate in the second round of the online survey.

The purpose of this survey is threefold. We will first ask your opinion on the additional recommendations. We will then zoom in on rating all recommendations. Finally, we will ask you to prioritise and rank the recommendations that you deem most important and this per tier of prevention (primary, secondary and tertiary). Finally we will ask you to provide information on the necessary conditions to implement a recommendation. The latter question is not mandatory, as we realise that for some recommendations this might be sector specific. 

We would like to point out that we take on board all feedback in the previous round. For reasons of comparability we decided not to change the wording of certain recommendations in this phase, however, we will integrate the received feedback on this aspect in the final report.

This second and last round of the survey is available until 7/05/2023.

There are 98 questions in this survey.

## 

### How strongly do you agree or disagree with the following statements? \*

Please choose the appropriate response for each item:

|  | Completely disagree | Disagree | Neither agree nor disagree | Agree | Completely agree |
| --- | --- | --- | --- | --- | --- |
| In the current circumstances, it is **feasible** to provide higher remuneration for prescribers for long follow up consultations dedicated to BZD/Z\*. |  |  |  |  |  |
| In the current circumstances, I would **support** the provision of higher remuneration for prescribers for long follow up consultations dedicated to BZD/Z\*. |  |  |  |  |  |

### If you would like to elaborate on your responses, please do so here (optional):

Please write your answer here:

### How important do you think it is to implement this recommendation? \*

Please choose the appropriate response for each item:

|  | Unimportant | Slightly important | Neither important nor unimportant | Important | Very Important |
| --- | --- | --- | --- | --- | --- |
| It is **important** to provide higher remuneration for prescribers for long follow up consultations dedicated to BZD/Z\*, |  |  |  |  |  |

Click here for more information on the meaning of the importance scale

### Are the conditions already met to make it feasible?  \*

Choose one of the following answers

Please choose **only one** of the following:

- Yes
- No
- I don't know

### What are the necessary conditions to make it feasible?

Only answer this question if the following conditions are met:

Answer was 'No' at question '4 [C2]' (Are the conditions already met to make it feasible? )

Please write your answer here:

## 

### How strongly do you agree or disagree with the following statements? \*

Please choose the appropriate response for each item:

|  | Completely disagree | Disagree | Neither agree nor disagree | Agree | Completely agree |
| --- | --- | --- | --- | --- | --- |
| In the current circumstances, it is **feasible** to give access to other healthcare professionals related to the treatment of BZD/Z\* to the part of the medical file related to prescriptions. |  |  |  |  |  |
| In the current circumstances, I would **support** giving access to other healthcare professionals related to the treatment of BZD/Z\* to the part of the medical file related to prescriptions. |  |  |  |  |  |

### If you would like to elaborate on your responses, please do so here (optional):

Please write your answer here:

### How important do you think it is to implement this recommendation? \*

Please choose the appropriate response for each item:

|  | Unimportant | Slightly important | Neither important nor unimportant | Important | Very Important |
| --- | --- | --- | --- | --- | --- |
| In the current circmstances, it is **important** to give access to other healthcare professionals related to the treatment of BZD/Z\* to the part of the medical file related to prescriptions. |  |  |  |  |  |

Click here for more information on the meaning of the importance scale

### Are the conditions already met to make it feasible?  \*

Choose one of the following answers

Please choose **only one** of the following:

- Yes
- No
- I don't know

### What are the necessary conditions to make it feasible?

Only answer this question if the following conditions are met:

Answer was 'No' at question '9 [C2]' (Are the conditions already met to make it feasible? )

Please write your answer here:

## 

### How strongly do you agree or disagree with the following statements? \*

Please choose the appropriate response for each item:

|  | Completely disagree | Disagree | Neither agree nor disagree | Agree | Completely agree |
| --- | --- | --- | --- | --- | --- |
| In the current circumstances, it is **feasible** to allow prescription/provision of BZD/Z\* per unit. |  |  |  |  |  |
| In the current circumstances, I would **support** allowing prescription/provision of BZD/Z\* per unit. |  |  |  |  |  |

### If you would like to elaborate on your responses, please do so here (optional):

Please write your answer here:

### How important do you think it is to implement this recommendation? \*

Please choose the appropriate response for each item:

|  | Unimportant | Slightly important | Neither important nor unimportant | Important | Very Important |
| --- | --- | --- | --- | --- | --- |
| In the current circmstances, it is **important** to allow prescription/provision of BZD/Z\* per unit. |  |  |  |  |  |

Click here for more information on the meaning of the importance scale

### Are the conditions already met to make it feasible?  \*

Choose one of the following answers

Please choose **only one** of the following:

- Yes
- No
- I don't know

### What are the necessary conditions to make it feasible?

Only answer this question if the following conditions are met:

Answer was 'No' at question '14 [C2]' (Are the conditions already met to make it feasible? )

Please write your answer here:

## 

### How strongly do you agree or disagree with the following statements? \*

Please choose the appropriate response for each item:

|  | Completely disagree | Disagree | Neither agree nor disagree | Agree | Completely agree |
| --- | --- | --- | --- | --- | --- |
| In the current circumstances, it is **feasible** to offer group therapy to non-hospitalised patients to support the tapering off process. |  |  |  |  |  |
| In the current circumstances, I would **support** offering group therapy to non-hospitalised patients to support the tapering off process. |  |  |  |  |  |

### If you would like to elaborate on your responses, please do so here (optional):

Please write your answer here:

### How important do you think it is to implement this recommendation? \*

Please choose the appropriate response for each item:

|  | Unimportant | Slightly important | Neither important nor unimportant | Important | Very Important |
| --- | --- | --- | --- | --- | --- |
| In the current circmstances, it is **important** to offer group therapy to non-hospitalised patients to support the tapering off process. |  |  |  |  |  |

Click here for more information on the meaning of the importance scale

### Are the conditions already met to make it feasible?  \*

Choose one of the following answers

Please choose **only one** of the following:

- Yes
- No
- I don't know

### What are the necessary conditions to make it feasible?

Only answer this question if the following conditions are met:

Answer was 'No' at question '19 [C2]' (Are the conditions already met to make it feasible? )

Please write your answer here:

## 

### How strongly do you agree or disagree with the following statements? \*

Please choose the appropriate response for each item:

|  | Completely disagree | Disagree | Neither agree nor disagree | Agree | Completely agree |
| --- | --- | --- | --- | --- | --- |
| In the current circumstances, it is **feasible** to undertake further research on the mechanisms surrounding the first prescription of BZD/Z\*. |  |  |  |  |  |
| In the current circumstances, I would **support** undertaking further research on the mechanisms surrounding the first prescription of BZD/Z\*. |  |  |  |  |  |

### If you would like to elaborate on your responses, please do so here (optional):

Please write your answer here:

### How important do you think it is to implement this recommendation? \*

Please choose the appropriate response for each item:

|  | Unimportant | Slightly important | Neither important nor unimportant | Important | Very Important |
| --- | --- | --- | --- | --- | --- |
| In the current circumstances, it is **important** to undertake further research on the mechanisms surrounding the first prescription of BZD/Z\*. |  |  |  |  |  |

Click here for more information on the meaning of the importance scale

### Are the conditions already met to make it feasible?  \*

Choose one of the following answers

Please choose **only one** of the following:

- Yes
- No
- I don't know

### What are the necessary conditions to make it feasible?

Only answer this question if the following conditions are met:

Answer was 'No' at question '24 [C2]' (Are the conditions already met to make it feasible? )

Please write your answer here:

## 

### How strongly do you agree or disagree with the following statements? \*

Please choose the appropriate response for each item:

|  | Completely disagree | Disagree | Neither agree nor disagree | Agree | Completely agree |
| --- | --- | --- | --- | --- | --- |
| In the current circumstances, it is **feasible** to create an inter-professional communication channel at local level, between pharmacists and GPs to discuss common patients. |  |  |  |  |  |
| In the current circumstances, I would **support** the creation an inter-professional communication channel at local level, between pharmacists and GPs to discuss common patients. |  |  |  |  |  |

### If you would like to elaborate on your responses, please do so here (optional):

Please write your answer here:

### How important do you think it is to implement this recommendation? \*

Please choose the appropriate response for each item:

|  | Unimportant | Slightly important | Neither important nor unimportant | Important | Very Important |
| --- | --- | --- | --- | --- | --- |
| In the current circmstances, it is **important** to create an inter-professional communication channel at local level, between pharmacists and GPs to discuss common patients. |  |  |  |  |  |

Click here for more information on the meaning of the importance scale

### Are the conditions already met to make it feasible?  \*

Choose one of the following answers

Please choose **only one** of the following:

- Yes
- No
- I don't know

### What are the necessary conditions to make it feasible?

Only answer this question if the following conditions are met:

Answer was 'No' at question '29 [C2]' (Are the conditions already met to make it feasible? )

Please write your answer here:

## 

### How strongly do you agree or disagree with the following statements? \*

Please choose the appropriate response for each item:

|  | Completely disagree | Disagree | Neither agree nor disagree | Agree | Completely agree |
| --- | --- | --- | --- | --- | --- |
| In the current circumstances, it is **feasible** to tailor specific residential addiction programmes to BZD/Z\*. |  |  |  |  |  |
| In the current circumstances, I would **support**  tailoring specific residential addiction programmes to BZD/Z\*. |  |  |  |  |  |

### If you would like to elaborate on your responses, please do so here (optional):

Please write your answer here:

### How important do you think it is to implement this recommendation? \*

Please choose the appropriate response for each item:

|  | Unimportant | Slightly important | Neither important nor unimportant | Important | Very Important |
| --- | --- | --- | --- | --- | --- |
| In the current circmstances, it is **important** to tailor specific residential addiction programmes to BZD/Z\*. |  |  |  |  |  |

Click here for more information on the meaning of the importance scale

### Are the conditions already met to make it feasible?  \*

Choose one of the following answers

Please choose **only one** of the following:

- Yes
- No
- I don't know

### What are the necessary conditions to make it feasible?

Only answer this question if the following conditions are met:

Answer was 'No' at question '34 [C2]' (Are the conditions already met to make it feasible? )

Please write your answer here:

## 

### How important do you think it is to implement this recommendation? \*

Please choose the appropriate response for each item:

|  | Unimportant | Slightly important | Neither important nor unimportant | Important | Very Important |
| --- | --- | --- | --- | --- | --- |
| In the current circmstances, it is **important** to implement an awareness raising campaign among the general public on tapering off BZD/Z\*. |  |  |  |  |  |

Click here for more information on the meaning of the importance scale

### Are the conditions already met to make it feasible?  \*

Choose one of the following answers

Please choose **only one** of the following:

- Yes
- No
- I don't know

### What are the necessary conditions to make it feasible?

Only answer this question if the following conditions are met:

Answer was 'No' at question '37 [C2]' (Are the conditions already met to make it feasible? )

Please write your answer here:

## 

### Dans quelle mesure pensez-vous qu’il est important de mettre en place cette recommandation ? \*

Please choose the appropriate response for each item:

|  | Unimportant | Slightly important | Neither important nor unimportant | Important | Very Important |
| --- | --- | --- | --- | --- | --- |
| In the current circumstances, it is **important** to implement an awareness raising campaign among the general public on tapering off benzodiazepines and Z-drugs. |  |  |  |  |  |

Click here for more information on the meaning of the importance scale

### Are the conditions already met to make it feasible?  \*

Choose one of the following answers

Please choose **only one** of the following:

- Yes
- No
- I don't know

### What are the necessary conditions to make it feasible?

Only answer this question if the following conditions are met:

Answer was 'No' at question '40 [C2]' (Are the conditions already met to make it feasible? )

Please write your answer here:

## 

### How important do you think it is to implement this recommendation? \*

Please choose the appropriate response for each item:

|  | Unimportant | Slightly important | Neither important nor unimportant | Important | Very Important |
| --- | --- | --- | --- | --- | --- |
| In the current circumstances, it is **important** to implement an awareness raising campaign among the general public on tapering off benzodiazepines and Z-drugs. |  |  |  |  |  |

Click here for more information on the meaning of the importance scale

### Are the conditions already met to make it feasible?  \*

Choose one of the following answers

Please choose **only one** of the following:

- Yes
- No
- I don't know

### What are the necessary conditions to make it feasible?

Only answer this question if the following conditions are met:

Answer was 'No' at question '43 [C2]' (Are the conditions already met to make it feasible? )

Please write your answer here:

## 

### How important do you think it is to implement this recommendation? \*

Please choose the appropriate response for each item:

|  | Unimportant | Slightly important | Neither important nor unimportant | Important | Very Important |
| --- | --- | --- | --- | --- | --- |
| In the current circumstances, it is **important** to implement an awareness raising campaign among the general public on tapering off benzodiazepines and Z-drugs. |  |  |  |  |  |

Click here for more information on the meaning of the importance scale

### Are the conditions already met to make it feasible?  \*

Choose one of the following answers

Please choose **only one** of the following:

- Yes
- No
- I don't know

### What are the necessary conditions to make it feasible?

Only answer this question if the following conditions are met:

Answer was 'No' at question '46 [C2]' (Are the conditions already met to make it feasible? )

Please write your answer here:

## 

### How important do you think it is to implement this recommendation? \*

Please choose the appropriate response for each item:

|  | Unimportant | Slightly important | Neither important nor unimportant | Important | Very Important |
| --- | --- | --- | --- | --- | --- |
| In the current circumstances, it is **important** to implement an awareness raising campaign among the general public on tapering off benzodiazepines and Z-drugs. |  |  |  |  |  |

Click here for more information on the meaning of the importance scale

### Are the conditions already met to make it feasible?  \*

Choose one of the following answers

Please choose **only one** of the following:

- Yes
- No
- I don't know

### What are the necessary conditions to make it feasible?

Only answer this question if the following conditions are met:

Answer was 'No' at question '49 [C2]' (Are the conditions already met to make it feasible? )

Please write your answer here:

## 

### How important do you think it is to implement this recommendation? \*

Please choose the appropriate response for each item:

|  | Unimportant | Slightly important | Neither important nor unimportant | Important | Very Important |
| --- | --- | --- | --- | --- | --- |
| In the current circumstances, it is **important** to implement an awareness raising campaign among the general public on tapering off benzodiazepines and Z-drugs. |  |  |  |  |  |

Click here for more information on the meaning of the importance scale

### Are the conditions already met to make it feasible?  \*

Choose one of the following answers

Please choose **only one** of the following:

- Yes
- No
- I don't know

### What are the necessary conditions to make it feasible?

Only answer this question if the following conditions are met:

Answer was 'No' at question '52 [C2]' (Are the conditions already met to make it feasible? )

Please write your answer here:

## 

### How important do you think it is to implement this recommendation? \*

Please choose the appropriate response for each item:

|  | Unimportant | Slightly important | Neither important nor unimportant | Important | Very Important |
| --- | --- | --- | --- | --- | --- |
| In the current circumstances, it is **important** to implement an awareness raising campaign among the general public on tapering off benzodiazepines and Z-drugs. |  |  |  |  |  |

Click here for more information on the meaning of the importance scale

### Are the conditions already met to make it feasible?  \*

Choose one of the following answers

Please choose **only one** of the following:

- Yes
- No
- I don't know

### What are the necessary conditions to make it feasible?

Only answer this question if the following conditions are met:

Answer was 'No' at question '55 [C2]' (Are the conditions already met to make it feasible? )

Please write your answer here:

## 

### How important do you think it is to implement this recommendation? \*

Please choose the appropriate response for each item:

|  | Unimportant | Slightly important | Neither important nor unimportant | Important | Very Important |
| --- | --- | --- | --- | --- | --- |
| In the current circumstances, it is **important** to implement an awareness raising campaign among the general public on tapering off benzodiazepines and Z-drugs. |  |  |  |  |  |

Click here for more information on the meaning of the importance scale

### Are the conditions already met to make it feasible?  \*

Choose one of the following answers

Please choose **only one** of the following:

- Yes
- No
- I don't know

### What are the necessary conditions to make it feasible?

Only answer this question if the following conditions are met:

Answer was 'No' at question '58 [C2]' (Are the conditions already met to make it feasible? )

Please write your answer here:

## 

### How important do you think it is to implement this recommendation? \*

Please choose the appropriate response for each item:

|  | Unimportant | Slightly important | Neither important nor unimportant | Important | Very Important |
| --- | --- | --- | --- | --- | --- |
| In the current circumstances, it is **important** to implement an awareness raising campaign among the general public on tapering off benzodiazepines and Z-drugs. |  |  |  |  |  |

Click here for more information on the meaning of the importance scale

### Are the conditions already met to make it feasible?  \*

Choose one of the following answers

Please choose **only one** of the following:

- Yes
- No
- I don't know

### What are the necessary conditions to make it feasible?

Only answer this question if the following conditions are met:

Answer was 'No' at question '61 [C2]' (Are the conditions already met to make it feasible? )

Please write your answer here:

## 

### How important do you think it is to implement this recommendation? \*

Please choose the appropriate response for each item:

|  | Unimportant | Slightly important | Neither important nor unimportant | Important | Very Important |
| --- | --- | --- | --- | --- | --- |
| In the current circumstances, it is **important** to implement an awareness raising campaign among the general public on tapering off benzodiazepines and Z-drugs. |  |  |  |  |  |

Click here for more information on the meaning of the importance scale

### Are the conditions already met to make it feasible?  \*

Choose one of the following answers

Please choose **only one** of the following:

- Yes
- No
- I don't know

### What are the necessary conditions to make it feasible?

Only answer this question if the following conditions are met:

Answer was 'No' at question '64 [C2]' (Are the conditions already met to make it feasible? )

Please write your answer here:

## 

### How important do you think it is to implement this recommendation? \*

Please choose the appropriate response for each item:

|  | Unimportant | Slightly important | Neither important nor unimportant | Important | Very Important |
| --- | --- | --- | --- | --- | --- |
| In the current circumstances, it is **important** to implement an awareness raising campaign among the general public on tapering off benzodiazepines and Z-drugs. |  |  |  |  |  |

Click here for more information on the meaning of the importance scale

### Are the conditions already met to make it feasible?  \*

Choose one of the following answers

Please choose **only one** of the following:

- Yes
- No
- I don't know

### What are the necessary conditions to make it feasible?

Only answer this question if the following conditions are met:

Answer was 'No' at question '67 [C2]' (Are the conditions already met to make it feasible? )

Please write your answer here:

## 

### How important do you think it is to implement this recommendation? \*

Please choose the appropriate response for each item:

|  | Unimportant | Slightly important | Neither important nor unimportant | Important | Very Important |
| --- | --- | --- | --- | --- | --- |
| In the current circumstances, it is **important** to implement an awareness raising campaign among the general public on tapering off benzodiazepines and Z-drugs. |  |  |  |  |  |

Click here for more information on the meaning of the importance scale

### Are the conditions already met to make it feasible?  \*

Choose one of the following answers

Please choose **only one** of the following:

- Yes
- No
- I don't know

### What are the necessary conditions to make it feasible?

Only answer this question if the following conditions are met:

Answer was 'No' at question '70 [C2]' (Are the conditions already met to make it feasible? )

Please write your answer here:

## 

### How important do you think it is to implement this recommendation? \*

Please choose the appropriate response for each item:

|  | Unimportant | Slightly important | Neither important nor unimportant | Important | Very Important |
| --- | --- | --- | --- | --- | --- |
| In the current circumstances, it is **important** to implement an awareness raising campaign among the general public on tapering off benzodiazepines and Z-drugs. |  |  |  |  |  |

Click here for more information on the meaning of the importance scale

### Are the conditions already met to make it feasible?  \*

Choose one of the following answers

Please choose **only one** of the following:

- Yes
- No
- I don't know

### What are the necessary conditions to make it feasible?

Only answer this question if the following conditions are met:

Answer was 'No' at question '73 [C2]' (Are the conditions already met to make it feasible? )

Please write your answer here:

## 

### How important do you think it is to implement this recommendation? \*

Please choose the appropriate response for each item:

|  | Unimportant | Slightly important | Neither important nor unimportant | Important | Very Important |
| --- | --- | --- | --- | --- | --- |
| In the current circumstances, it is **important** to implement an awareness raising campaign among the general public on tapering off benzodiazepines and Z-drugs. |  |  |  |  |  |

Click here for more information on the meaning of the importance scale

### Are the conditions already met to make it feasible?  \*

Choose one of the following answers

Please choose **only one** of the following:

- Yes
- No
- I don't know

### What are the necessary conditions to make it feasible?

Only answer this question if the following conditions are met:

Answer was 'No' at question '76 [C2]' (Are the conditions already met to make it feasible? )

Please write your answer here:

## 

### How important do you think it is to implement this recommendation? \*

Please choose the appropriate response for each item:

|  | Unimportant | Slightly important | Neither important nor unimportant | Important | Very Important |
| --- | --- | --- | --- | --- | --- |
| In the current circumstances, it is **important** to implement an awareness raising campaign among the general public on tapering off benzodiazepines and Z-drugs. |  |  |  |  |  |

Click here for more information on the meaning of the importance scale

### Are the conditions already met to make it feasible?  \*

Choose one of the following answers

Please choose **only one** of the following:

- Yes
- No
- I don't know

### What are the necessary conditions to make it feasible?

Only answer this question if the following conditions are met:

Answer was 'No' at question '79 [C2]' (Are the conditions already met to make it feasible? )

Please write your answer here:

## 

### How important do you think it is to implement this recommendation? \*

Please choose the appropriate response for each item:

|  | Unimportant | Slightly important | Neither important nor unimportant | Important | Very Important |
| --- | --- | --- | --- | --- | --- |
| In the current circumstances, it is **important** to implement an awareness raising campaign among the general public on tapering off benzodiazepines and Z-drugs. |  |  |  |  |  |

Click here for more information on the meaning of the importance scale

### Are the conditions already met to make it feasible?  \*

Choose one of the following answers

Please choose **only one** of the following:

- Yes
- No
- I don't know

### What are the necessary conditions to make it feasible?

Only answer this question if the following conditions are met:

Answer was 'No' at question '82 [C2]' (Are the conditions already met to make it feasible? )

Please write your answer here:

## 

### How important do you think it is to implement this recommendation? \*

Please choose the appropriate response for each item:

|  | Unimportant | Slightly important | Neither important nor unimportant | Important | Very Important |
| --- | --- | --- | --- | --- | --- |
| In the current circumstances, it is **important** to implement an awareness raising campaign among the general public on tapering off benzodiazepines and Z-drugs. |  |  |  |  |  |

Click here for more information on the meaning of the importance scale

### Are the conditions already met to make it feasible?  \*

Choose one of the following answers

Please choose **only one** of the following:

- Yes
- No
- I don't know

### What are the necessary conditions to make it feasible?

Only answer this question if the following conditions are met:

Answer was 'No' at question '85 [C2]' (Are the conditions already met to make it feasible? )

Please write your answer here:

## 

### How important do you think it is to implement this recommendation? \*

Please choose the appropriate response for each item:

|  | Unimportant | Slightly important | Neither important nor unimportant | Important | Very Important |
| --- | --- | --- | --- | --- | --- |
| In the current circumstances, it is **important** to create an ombudsperson for healthcare practitioners to report other practitioners who over-prescribe, prescribe or delivered unsafely BZD/Z\*. |  |  |  |  |  |

Click here for more information on the meaning of the importance scale

### Are the conditions already met to make it feasible?  \*

Choose one of the following answers

Please choose **only one** of the following:

- Yes
- No
- I don't know

### What are the necessary conditions to make it feasible?

Only answer this question if the following conditions are met:

Answer was 'No' at question '88 [C2]' (Are the conditions already met to make it feasible? )

Please write your answer here:

## 

### How important do you think it is to implement this recommendation? \*

Please choose the appropriate response for each item:

|  | Unimportant | Slightly important | Neither important nor unimportant | Important | Very Important |
| --- | --- | --- | --- | --- | --- |
| In the current circumstances, it is **important** to extend the patient inclusion criteria of the new reimbursement scheme for the compounding of smaller doses of BZD/Z\* to residents living in nursing homes. |  |  |  |  |  |

Click here for more information on the meaning of the importance scale

### Are the conditions already met to make it feasible?  \*

Choose one of the following answers

Please choose **only one** of the following:

- Yes
- No
- I don't know

### What are the necessary conditions to make it feasible?

Only answer this question if the following conditions are met:

Answer was 'No' at question '91 [C2]' (Are the conditions already met to make it feasible? )

Please write your answer here:

## 

### How important do you think it is to implement this recommendation? \*

Please choose the appropriate response for each item:

|  | Unimportant | Slightly important | Neither important nor unimportant | Important | Very Important |
| --- | --- | --- | --- | --- | --- |
| In the current circumstances, it is **important** to implement an awareness raising campaign among the general public on tapering off benzodiazepines and Z-drugs. |  |  |  |  |  |

Click here for more information on the meaning of the importance scale

### Are the conditions already met to make it feasible?  \*

Choose one of the following answers

Please choose **only one** of the following:

- Yes
- No
- I don't know

### What are the necessary conditions to make it feasible?

Only answer this question if the following conditions are met:

Answer was 'No' at question '94 [C2]' (Are the conditions already met to make it feasible? )

Please write your answer here:

## 

### In the current circumstances, which are the three most important recommendations to implement in the primary prevention? Select 3 and order them with the most important first:

All your answers must be different and you must rank in order.

Please select 3 answers

Please number each box in order of preference from 1 to 6

Please choose at least 3 items.

Please choose no more than 3 items.

- Implementing an awareness raising campaign among the general public on tapering off benzodiazepines and Z-drugs.
- Implementing an awareness raising campaign for patients on the challenges of withdrawing benzodiazepines and Z-drugs from multiple medications.
- Implementing an awareness raising campaign for professionals on the challenges of withdrawing from multiple medications.
- An awareness raising campaign of the risks of benzodiazepines and Z-drugs in empathetic and non-stigmatising way.
- Adding warnings of the risk of dependance on the benzodiazepines and Z-drugs package.
- Undertake further research on the mechanisms surrounding the first prescription of benzodiazepines and Z-drugs.

### In the current circumstances, which are the three most important recommendations to implement in the secondary prevention? Select 3 and order them with the most important first:

All your answers must be different and you must rank in order.

Please select 3 answers

Please number each box in order of preference from 1 to 7

Please choose at least 3 items.

Please choose no more than 3 items.

- Increase the price per benzodiazepines and Z-drugs package.
- Create smaller packages of benzodiazepines and Z-drugs.
- Provide information by the prescriber to the patient regarding the risks of dependency of benzodiazepines and Z-drugs at first use.
- Provide higher remuneration for prescribers for long follow up consultations dedicated to benzodiazepines and Z-drugs\*.
- Give access to other BZD prescribers/providers to the part of the medical file related to prescriptions.
- Allow the carer to dispense one or two doses of benzodiazepines and Z-drugs\* at the same time to provide the correct dose.
- Create an inter-professional communication channel at local level, between pharmacists and GPs to discuss common patients.

### In the current circumstances, which are the three most important recommendations to implement in the tertiary prevention? Select 3 and order them with the most important first:

Please select 5 answers

Please number each box in order of preference from 1 to 14

Please choose at least 5 items.

Please choose no more than 5 items.

- Encourage prescribers to add the indication for substance use disorders next to insomnia/anxiety to patient records when use exceeds guidelines.
- The establishment of an agreement between the prescriber, the pharmacist and the patient to keep the same prescriber and pharmacist throughout treatment.
- Creating a shared policy position between different professionals groups in addiction care concerning the management of benzodiazepines and Z-drugs.
- Implementing a training course on difficult tapering-off processes related to benzodiazepines and Z-drugs for professionals.
- Establish and providing a list of healthcare providers specialised in tapering off of benzodiazepines and Z-drugs.
- Establish a support and advice line for people who want to taper off of benzodiazepines and Z-drugs.
- Develop a ‘Benzo-buddy’ system.
- Share patient testimonials about benzodiazepines and Z-drugs tapering-off.
- Develop culturally appropriate patient materials.
- Create an ombudsperson for healthcare practitioners to report other practitioners who over-prescribe, prescribe or delivered unsafely benzodiazepines and Z-drugs.
- Extend the patient inclusion criteria of the new reimbursement scheme for the compounding of smaller doses of benzodiazepines and Z-drugs to residents living in nursing homes.
- Extend the patient inclusion criteria of the new reimbursement scheme for the compounding of smaller doses of benzodiazepines and Z-drugs to patients who are taking more than one type of benzodiazepines or Z-drugs.
- Offer group therapy to ambulant patients tu support the tapering off process.
- Tailor residential addiction care programmes, specifically to benzodiazepines and Z-drugs withdrawal.

Thank you for your participation to this study!

If you have any questions, feel free to email us at dumg@uliege.be

  
09.05.2023 – 00:00  
  
Submit your survey.  
Thank you for completing this survey.
