# Supplementary material for: Primary, secondary and tertiary prevention of long-term benzodiazepine receptor agonists use in Belgium: a policy Delphi
Source: Arch Public Health. 2025 Jun 23;83:164. doi: 10.1186/s13690-025-01580-w (PMC12183905; doi:10.1186/s13690-025-01580-w)
Supplement: Supplementary file 5 — Additional file 5. [file 13690_2025_1580_MOESM5_ESM.docx]

| **Feasibility** | | | | | |
| --- | --- | --- | --- | --- | --- |
| **Statement number** | **Completely agree   (%)** | **Agree**  **(%)** | **Neither agree nor disagree**  **(%)** | **Disagree**  **(%)** | **Completely disagree**  **(%)** |
| Q1 | 45,05 | 45,05 | 6,30 | 2,70 | 0,90 |
| Q2 | 36,94 | 47,75 | 7,20 | 6,31 | 1,80 |
| Q3 | 54,95 | 34,23 | 7,21 | 1,80 | 1,80 |
| Q4 | 52,25 | 26,13 | 16,22 | 3,60 | 1,80 |
| Q5 | 55,86 | 26,13 | 9,91 | 7,21 | 0,90 |
| Q6 | 38,71 | 46,77 | 11,29 | 1,61 | 1,61 |
| Q7 | 7,21 | 18,02 | 29,73 | 26,13 | 18,92 |
| Q8 | 50,45 | 26,13 | 14,41 | 4,50 | 4,50 |
| Q9 | 63,06 | 27,93 | 6,31 | 1,80 | 0,90 |
| Q10 | 8,06 | 27,42 | 53,23 | 8,06 | 3,23 |
| Q11 | 16,13 | 38,71 | 30,65 | 9,68 | 4,84 |
| Q12 | 32,26 | 35,48 | 22,58 | 8,06 | 1,61 |
| Q13 | 30,63 | 30,63 | 26,13 | 7,21 | 5,41 |
| Q14 | 27,03 | 27,03 | 24,32 | 16,22 | 5,41 |
| Q15 | 27,93 | 44,14 | 21,62 | 4,50 | 1,80 |
| Q16 | 24,19 | 48,39 | 20,97 | 4,84 | 1,61 |
| Q17 | 51,35 | 39,64 | 8,11 | 0,00 | 0,90 |
| Q18 | 27,93 | 42,34 | 21,62 | 7,21 | 0,90 |
| Q19 | 37,84 | 31,53 | 21,62 | 7,21 | 1,80 |
| Q20 | 17,12 | 34,23 | 36,04 | 9,91 | 2,70 |
| Q21 | 34,23 | 49,55 | 8,11 | 6,31 | 1,80 |
| Q22 | 40,54 | 39,64 | 14,41 | 2,70 | 2,70 |
| Q23 | 18,02 | 21,62 | 29,73 | 18,02 | 12,61 |
| Q24 | 30,63 | 32,43 | 27,03 | 5,41 | 4,50 |
| Q25 | 31,53 | 34,23 | 28,83 | 4,50 | 0,90 |
| Q26 | 30,65 | 46,77 | 16,13 | 4,84 | 1,61 |
| Q27 | 20,97 | 43,55 | 29,03 | 6,45 | 0,00 |

**Additional file 5**

| **Support** | | | | | |
| --- | --- | --- | --- | --- | --- |
| **Statement number** | **Completely agree**  **(%)** | **Agree**  **(%)** | **Neither agree nor disagree**  **(%)** | **Disagree**  **(%)** | **Completely disagree**  **(%)** |
| Q1 | 59,46 | 29,73 | 5,41 | 3,60 | 1,80 |
| Q2 | 53,15 | 36,94 | 6,31 | 1,80 | 1,80 |
| Q3 | 64,86 | 27,03 | 7,21 | 0,00 | 0,90 |
| Q4 | 63,96 | 22,52 | 8,11 | 4,50 | 0,90 |
| Q5 | 63,96 | 22,52 | 8,11 | 4,50 | 0,90 |
| Q6 | 41,94 | 38,71 | 14,52 | 1,61 | 3,23 |
| Q7 | 6,31 | 7,21 | 28,83 | 29,73 | 27,93 |
| Q8 | 56,76 | 18,02 | 15,32 | 2,70 | 7,21 |
| Q9 | 75,68 | 20,72 | 2,70 | 0,00 | 0,90 |
| Q10 | 19,35 | 35,48 | 24,19 | 16,13 | 4,84 |
| Q11 | 30,65 | 50,00 | 8,06 | 8,06 | 3,23 |
| Q12 | 50,00 | 30,65 | 16,13 | 1,61 | 1,61 |
| Q13 | 34,23 | 26,13 | 22,52 | 9,91 | 7,21 |
| Q14 | 37,84 | 25,23 | 17,12 | 14,41 | 5,41 |
| Q15 | 41,44 | 44,14 | 11,71 | 0,90 | 1,80 |
| Q16 | 38,71 | 41,94 | 9,68 | 4,84 | 4,84 |
| Q17 | 61,26 | 33,33 | 4,50 | 0,00 | 0,90 |
| Q18 | 45,05 | 34,23 | 13,51 | 6,31 | 0,90 |
| Q19 | 51,35 | 24,32 | 18,02 | 4,50 | 1,80 |
| Q20 | 28,83 | 38,74 | 24,32 | 5,41 | 2,70 |
| Q21 | 43,24 | 42,34 | 9,01 | 1,80 | 3,60 |
| Q22 | 54,95 | 33,33 | 8,11 | 1,80 | 1,80 |
| Q23 | 25,23 | 18,92 | 27,03 | 12,61 | 16,22 |
| Q24 | 45,05 | 27,03 | 21,62 | 1,80 | 4,50 |
| Q25 | 42,34 | 33,33 | 18,92 | 4,50 | 0,90 |
| Q26 | 40,32 | 51,61 | 8,06 | 0,00 | 0,00 |
| Q27 | 38,71 | 35,48 | 17,74 | 8,06 | 0,00 |

| **Importance** | | | | | |
| --- | --- | --- | --- | --- | --- |
| **Statement number** | **Very important**  **(%)** | **Important**  **(%)** | **Neither important nor unimportant**  **(%)** | **Slightly important**  **(%)** | **Unimportant**  **(%)** |
| Q1 | 59,68 | 32,26 | 6,45 | 0,00 | 1,61 |
| Q2 | 53,23 | 40,32 | 4,84 | 1,61 | 0,00 |
| Q3 | 53,23 | 35,48 | 9,68 | 1,61 | 0,00 |
| Q4 | 66,13 | 32,26 | 1,61 | 0,00 | 0,00 |
| Q5 | 54,84 | 20,97 | 12,90 | 8,06 | 3,23 |
| Q6 | 38,71 | 41,94 | 11,29 | 6,45 | 1,61 |
| Q7 | 4,84 | 8,06 | 29,03 | 20,97 | 37,10 |
| Q8 | 48,39 | 33,87 | 9,68 | 4,84 | 3,23 |
| Q9 | 74,19 | 24,19 | 0,00 | 1,61 | 0,00 |
| Q10 | 24,19 | 37,10 | 19,35 | 9,68 | 9,68 |
| Q11 | 25,81 | 45,16 | 16,13 | 8,06 | 4,84 |
| Q12 | 32,26 | 41,94 | 14,52 | 8,06 | 3,23 |
| Q13 | 22,58 | 38,71 | 24,19 | 9,68 | 4,84 |
| Q14 | 24,19 | 37,10 | 19,35 | 9,68 | 9,68 |
| Q15 | 35,48 | 51,61 | 12,90 | 0,00 | 0,00 |
| Q16 | 30,65 | 50,00 | 11,29 | 1,61 | 6,45 |
| Q17 | 51,61 | 43,55 | 3,23 | 1,61 | 0,00 |
| Q18 | 37,10 | 40,32 | 16,13 | 4,84 | 1,61 |
| Q19 | 33,87 | 35,48 | 20,97 | 6,45 | 3,23 |
| Q20 | 19,35 | 38,71 | 33,87 | 4,84 | 3,23 |
| Q21 | 35,48 | 46,77 | 11,29 | 6,45 | 0,00 |
| Q22 | 54,84 | 37,10 | 8,06 | 0,00 | 0,00 |
| Q23 | 24,19 | 20,97 | 22,58 | 12,90 | 19,35 |
| Q24 | 43,55 | 43,55 | 11,29 | 1,61 | 0,00 |
| Q25 | 41,94 | 48,39 | 8,06 | 1,61 | 0,00 |
| Q26 | 30,65 | 50,00 | 11,29 | 1,61 | 6,45 |
| Q27 | 37,10 | 30,65 | 22,58 | 3,23 | 6,45 |

| **Conditions** | | | |
| --- | --- | --- | --- |
| **Statement number** | **Yes**  **(%)** | **No**  **(%)** | **I don't Know**  **(%)** |
| Q1 | 66,13 | 25,81 | 8,06 |
| Q2 | 64,52 | 29,03 | 6,45 |
| Q3 | 72,58 | 20,97 | 6,45 |
| Q4 | 67,74 | 25,81 | 6,45 |
| Q5 | 62,90 | 27,42 | 9,68 |
| Q6 | 59,68 | 32,26 | 8,06 |
| Q7 | 37,10 | 43,55 | 19,35 |
| Q8 | 61,29 | 30,65 | 8,06 |
| Q9 | 69,35 | 22,58 | 8,06 |
| Q10 | 46,77 | 38,71 | 14,52 |
| Q11 | 30,65 | 53,23 | 16,13 |
| Q12 | 64,52 | 25,81 | 9,68 |
| Q13 | 38,71 | 43,55 | 17,74 |
| Q14 | 43,55 | 40,32 | 16,13 |
| Q15 | 20,97 | 59,68 | 19,35 |
| Q16 | 53,23 | 37,10 | 9,68 |
| Q17 | 64,52 | 25,81 | 9,68 |
| Q18 | 38,71 | 43,55 | 17,74 |
| Q19 | 43,55 | 40,32 | 16,13 |
| Q20 | 20,97 | 59,68 | 19,35 |
| Q21 | 53,23 | 37,10 | 9,68 |
| Q22 | 53,23 | 33,87 | 12,90 |
| Q23 | 17,74 | 50,00 | 32,26 |
| Q24 | 37,10 | 46,77 | 16,13 |
| Q25 | 46,77 | 46,77 | 6,45 |
| Q26 | 43,55 | 37,10 | 19,35 |
| Q27 | 33,87 | 48,39 | 17,74 |
